# Optimal Composition of Li Argyrodite with Harmonious Conductivity and Chemical/Electrochemical Stability: Fine‐Tuned Via Tandem Particle Swarm Optimization

**DOI:** 10.1002/advs.202201648

**Published:** 2022-07-21

**Authors:** Sunggeun Shim, Woon Bae Park, Jungmin Han, Jinhyeok Lee, Byung Do Lee, Jin‐Woong Lee, Jung Yong Seo, S. J. Richard Prabakar, Su Cheol Han, Satendra Pal Singh, Chan‐Cuk Hwang, Docheon Ahn, Sangil Han, Kyusung Park, Kee‐Sun Sohn, Myoungho Pyo

**Affiliations:** ^1^ Faculty of Nanotechnology and Advanced Materials Engineering Sejong University Seoul 05006 Republic of Korea; ^2^ Department of Advanced Components and Materials Engineering Sunchon National University Chonnam 57922 Republic of Korea; ^3^ Next Generation Development Team Samsung SDI R&D Center Suwon 16678 Republic of Korea; ^4^ Beamline Department Pohang Accelerator Laboratory Pohang 790‐784 Republic of Korea

**Keywords:** all‐solid‐state battery, argyrodite, ionic conductivity, particle swarm optimization, solid‐state electrolyte

## Abstract

A tandem (two‐step) particle swarm optimization (PSO) algorithm is implemented in the argyrodite‐based multidimensional composition space for the discovery of an optimal argyrodite composition, i.e., with the highest ionic conductivity (7.78 mS cm^−1^). To enhance the industrial adaptability, an elaborate pellet preparation procedure is not used. The optimal composition (Li_5.5_PS_4.5_Cl_0.89_Br_0.61_) is fine‐tuned to enhance its practical viability by incorporating oxygen in a stepwise manner. The final composition (Li_5.5_PS_4.23_O_0.27_Cl_0.89_Br_0.61_), which exhibits an ionic conductivity (*σ*_ion_) of 6.70 mS cm^−1^ and an activation barrier of 0.27 eV, is further characterized by analyzing both its moisture and electrochemical stability. Relative to the other compositions, the exposure of Li_5.5_PS_4.23_O_0.27_Cl_0.89_Br_0.61_ to a humid atmosphere results in the least amount of H_2_S released and a negligible change in structure. The improvement in the interfacial stability between the Li(Ni_0.9_Co_0.05_Mn_0.05_)O_2_ cathode and Li_5.5_PS_4.23_O_0.27_Cl_0.89_Br_0.61_ also results in greater specific capacity during fast charge/discharge. The structural and chemical features of Li_5.5_PS_4.5_Cl_0.89_Br_0.61_ and Li_5.5_PS_4.23_O_0.27_Cl_0.89_Br_0.61_ argyrodites are characterized using synchrotron X‐ray diffraction, Raman spectroscopy, and X‐ray photoelectron spectroscopy. This work presents a novel argyrodite composition with favorably balanced properties while providing broad insights into material discovery methodologies with applications for battery development.

## Graphical abstract

A two‐step optimization algorithm (tandem PSO) is applied to rapidly identify the argyrodite composition with the highest attainable *σ*_ion_ in a multidimensional search space. The composition of the PSO‐determined optimal sample composition is further tuned to enhance its chemical/electrochemical stability. The identified argyrodite exhibits enhanced stability against moisture and high voltage and, as an all‐solid‐state battery, would deliver higher capacities, particularly during fast charge–discharge cycles.

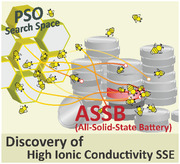

## 1 Introduction

Since the first report on the unusually high Li^+^ mobility of Li_6_PS_5_X (X = Cl and Br) by Deiseroth et al. in 2008,^[^ ^1^ ^]^ Li argyrodites have received considerable attention as a promising solid‐state electrolyte (SSE) for all‐solid‐state lithium batteries.^[^ ^2^, ^3^, ^4^, ^5^ ^]^ Various theoretical^[^ ^6^, ^7^, ^8^, ^9^ ^]^ and experimental^[^ ^10^, ^11^, ^12^, ^13^, ^14^, ^15^, ^16^, ^17^, ^18^ ^]^ investigations immediately followed to identify the origin of the high ionic conductivities of Li_6_PS_5_Cl and Li_6_PS_5_Br (10^−3^–10^−2^ S cm^−1^)^[^ ^12^, ^15^, ^18^ ^]^ in contrast to the insulating character of Li_6_PS_5_I (≈10^−7^–10^−6^ S cm^−1^).^[^ ^11^, ^14^, ^18^ ^]^ As a result, it is now well known that Li^+^ conduction is influenced by the distribution of S^2−^/X^−^ and Li^+^. The degree of anion disorder between two crystallographic sites (4a and 4d) is believed to be crucial for high conductivities, which decreases (i.e., becomes more ordered) when the S^2−^/X^−^ size mismatch is large (as in Li_6_PS_5_I).^[^ ^1^, ^7^, ^8^, ^14^ ^]^ An optimal Cl^−^ distribution of 1:3 over the 4a and 4d sites has been reported for the facile migration of Li^+^ between cages (inter‐cage jump) and thus, high ionic conduction.^[^ ^8^, ^19^, ^20^ ^]^ The Li^+^ occupancy at 24 g and 48 h sites has also been shown to affect ion transport.^[^ ^10^, ^18^ ^]^ It was recently reported that the increased Li^+^ occupancy at 24 g sites (transition state sites on the 48–48 h diffusion pathways) could promote ionic conduction,^[^ ^21^ ^]^ although independent control of the 24 g occupancy was not possible because the distribution of Li^+^ could also be influenced by the anion disorder.^[^ ^22^ ^]^

Soon after these pioneering works, various modifications of pristine Li_6_PS_5_X were executed to further increase the ionic conductivity of argyrodites. With the exception of a few attempts to substitute P^5+^ with tetravalent cations,^[^ ^23^, ^24^, ^25^, ^26^, ^27^, ^28^ ^]^ most efforts have focused on the substitution of S^2−^ by more polarizable anions (Se^2−^ and Te^2−^)^[^ ^29^, ^30^, ^31^ ^]^ and fine‐tuning the relative compositions of S^2−^/X^−^ and/or Cl^−^/Br^−^.^[^ ^32^, ^33^, ^34^, ^35^, ^36^, ^37^ ^]^ However, the notion of “the softer the lattice, the better it is” has not always held true.^[^ ^18^, ^32^ ^]^ Bernges et al. reported that an increase of “*x*” in Li_6_PS_5−_ *_x_* Se*_x_* Br resulted in negligible changes in *σ*_ion_.^[^ ^31^ ^]^ They claimed that the enhancement of the lattice softness also induces the reduction of the anion site disorder, which eventually diminishes the beneficial effect of substitutions. In addition, high Li content has been reported to enhance the ionic conductivity at room temperature.^[^ ^38^, ^39^, ^40^ ^]^

Despite these contributions, the prediction of ionic conductivity as a function of the structure/composition of argyrodites is still not straightforward because diverse synthetic procedures and various methods of measurement may yield over‐ or underestimated conductivity values. These problems have been exemplified in studies on the determination of the optimal Cl content in L_6−_ *_x_* PS_5−_ *_x_* Cl_1+_ *_x_*. While the highest conductivity values of 9.4–10.2 mS cm^−1^ were reported for *x* = 0.5 (Li_5.5_PS_4.5_Cl_1.5_) by two groups independently,^[^ ^33^, ^34^ ^]^ Li_5.7_PS_4.7_Cl_1.3_ and Li_5.3_PS_4.3_Cl_1.7_ have been shown by other groups to exhibit the highest room‐temperature ionic conductivity (*σ*_RT_) of 6.4 and 24 mS cm^−1^, respectively.^[^ ^22^, ^35^ ^]^ The conductivity values reported even for stoichiometric L_6_PS_5_X are also widely scattered from one research group to another. The *σ*_RT_ values of Li_6_PS_5_Cl and Li_6_PS_5_Br presented to date have ranged from 0.033 to 4.96 (0.033,^[^ ^12^, ^14^ ^]^ 0.79,^[^ ^32^ ^]^ 1.1,^[^ ^18^ ^]^ 1.18,^[^ ^17^ ^]^ 1.33,^[^ ^15^ ^]^ 2.4,^[^ ^41^ ^]^ 2.5,^[^ ^33^ ^]^ 3.15,^[^ ^42^ ^]^ 4.96^[^ ^43^ ^]^) and 0.032 to 5.5 mS cm^−1^ (0.032,^[^ ^12^, ^14^ ^]^ 0.19,^[^ ^44^ ^]^ 0.36,^[^ ^32^ ^]^ 0.7,^[^ ^24^ ^]^ 1.0,^[^ ^18^ ^]^ 1.2,^[^ ^45^ ^]^ 1.38,^[^ ^46^ ^]^ 1.9,^[^ ^41^ ^]^ 2.58,^[^ ^47^ ^]^ 2.8,^[^ ^31^ ^]^ 3.1,^[^ ^48^ ^]^ 5.5^[^ ^21^ ^]^), respectively. These variations are most likely because the anion disorder and Li^+^ distribution are very sensitive to the synthesis procedure (solution synthesis,^[^ ^41^, ^44^, ^48^ ^]^ ball‐milling conditions,^[^ ^36^, ^46^, ^49^ ^]^ and sintering temperature/time^[^ ^42^, ^43^, ^47^ ^]^). Pellet preparation conditions (time, pressure, and temperature) can also significantly affect the final measurements.^[^ ^50^ ^]^

In this study, we propose a facile strategy to identify new compounds with specific target properties (an argyrodite with a relatively high *σ*_ion_, moisture resistivity and interfacial stability). We first implemented tandem particle swarm optimization (PSO)^[^ ^51^ ^]^ to arrive at an argyrodite composition with the highest‐possible ionic conductivity at room temperature. To rapidly reach one of the local maxima or global maximum, *σ*_ion_ is adopted as a single objective function rather than simultaneously optimizing multiple objective functions. The *σ*_ion_ was determined for a pellet fabricated using a nonelaborate procedure to increase its viability for industrial application. We then modified the composition of the PSO‐nominated optimum argyrodite to introduce moisture resistivity and interfacial stability. The final argyrodite composition with balanced properties was identified; its structural and electrochemical features were subsequently characterized. We hope that this work will provide broad insights into the discovery of novel materials for battery fabrication.

## 2 Results and Discussion

### 2.1 PSO Algorithm

PSO is a population‐based metaheuristic approach,^[^ ^51^ ^]^ which is one of the most widely used metaheuristics (along with the genetic algorithm).^[^ ^52^ ^]^ PSO is a zero‐order noncalculus‐based method (i.e., no gradients are needed) that works best in an inherently continuous decision (design) variable space. The conceptual origin of PSO is based on the social behavior of a swarm. The decision variable space is regarded as a field wherein the swarm moves and locations full of flower nectar are regarded as local (or global) optima. PSO starts by assigning completely random positions and velocities to every particle (individual) in the swarm. The objective functions are evaluated for each individual and the new locations of the individuals in the swarm are determined by a scaled sum of three velocity vectors: the inertial vector of an individual of interest; a vector from the current position of the individual to the current global best position; and a vector from the current position of the individual to the best position that each individual has ever visited in its past trajectory. As the PSO iteration proceeds, the swarm becomes congested and a major portion of individuals eventually converges to a restricted area (exploitation), which is presumed to be the global optimum, while several outliers are located far away from the converged area (exploration). Thus, the optimal point which maximizes the objective function is discovered in later rounds within a preset search space. We recently employed PSO for the discovery of novel inorganic functional materials^[^ ^53^, ^54^, ^55^, ^56^, ^57^, ^58^, ^59^ ^]^ and established a so‐called PSO‐involved material discovery system.

The social behavior of a swarm is a key concept for PSO. Every individual in the swarm communicates with others to achieve a common goal. Every argyrodite, with a specific composition and processing conditions, located in the search space represents an individual in the swarm. A total of 20 compositions were randomly determined initially, and each of them was run through the search space by a weighted sum of three velocity vectors. The first vector points at the instantaneous best individual in the swarm, which means that we had to compare all the argyrodite samples in terms of the Li ionic conductivity (i.e., the objective function) at every step of the PSO and determine the individual with the highest value. The second velocity vector points toward the best location acquired in the past trajectory of each individual, which implies that every individual in the swarm memorizes its own best location, using its past trajectory that it has ever passed through. Finally, the last velocity vector is an inertia term initialized as a random vector in the first swarm.

The way in which PSO was executed in the present investigation differs sharply from conventional PSO‐based approaches. It should be noted that the aim of PSO in the present study was not a typical model parameter evaluation. The object function evaluation was obtained through the actual material synthesis and characterization. Namely, the objective function at every step of the PSO was evaluated via the real‐world argyrodite synthesis and subsequent ionic conductivity measurement. Thus, our approach resembles a PSO‐assisted experimental design rather than the typical PSO‐based parameter evaluation. A conventional PSO needs to be iterated thousands of times to attain a complete convergence to the global optimum. In sharp contrast to this PSO routine, PSO processes in the present study were terminated after the fifth round because of the high‐cost experimentation‐based evaluation of the objective functions. Nonetheless, a limited number of iterations up to, at best, the fifth round are acceptable because the major part of the improvement is always realized during the several initial iterations for all the metaheuristic‐based optimization algorithms. The main goal of PSO in the present study was not to achieve complete convergence on the global optimum but to nominate a greater number of plausible argyrodite candidates than other knowledge‐based trial‐and‐error approaches, in other words, our PSO execution resulted in preliminary, rough guidance rather than complete optimization.

### 2.2 Search Space Design and Tandem PSO Execution for Higher *σ*_ion_

Single‐objective PSO was adopted in the present study (the objective function is determined as *σ*_ion_ at room temperature). The decision variables were the composition and processing conditions of the search space. A set of argyrodite samples selected for each round was synthesized and the *σ*_ion_ values of the individual samples were evaluated. The first PSO round began with 20 argyrodite samples chosen randomly, followed by consecutive rounds governed by the PSO algorithm.

**Figure 1:**
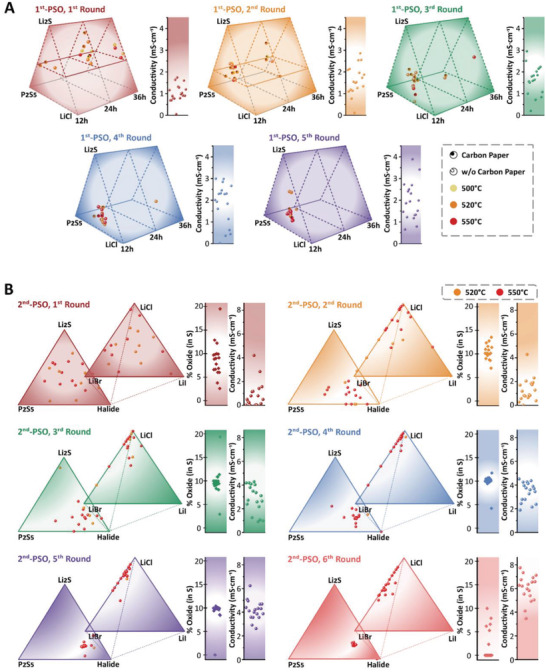
Schematic of the search space (decision variable space) and *σ*_ion_ results from the tandem PSO. A) The basic argyrodite compositional search space in the first PSO execution. Note that the apex composition of the ternary system is actually Li_9_PS_6.5_Cl‐Li_2.1_PS_3.39_Cl_0.31_‐Li_9_PS_5.39_Cl_3.23_, although it is represented as Li_2_S‐Li_2_P_5_‐LiCl for brevity. All individuals in each generation (round) are schematically represented in the trigonal‐shaped search space. Two processing variables (temperature and synthesis additive) have also been included. The *σ*_ion_ values for five rounds are plotted on the right side of the corresponding swarm (the horizontal distribution has been introduced for clarity and has no physical significance). B) The second PSO execution in the multidoped argyrodite compositional search space. Note that the actual apex composition of the ternary system is Li_6.14_PS_4.89_X_1.36_–Li_3.77_PS_3.93_X_0.9_–Li_6.15_PS_4.65_X_1.86_, X = halides, although it is represented as Li_2_S‐Li_2_P_5_‐Halide for brevity. All individuals in each round are schematically represented in two ternary and one unary search spaces. The *σ*_ion_ values for five rounds are also represented along the rightmost vertical axis. The decision variables (composition, firing temperature, and carbon paper) and objective function (Li ionic conductivity) values for every generation are given in Table S3 (Supporting Information).

A tandem (two‐step) PSO‐driven composition fine‐tuning procedure was implemented. The first PSO execution is restricted to typical composition ranges under a certain range of processing conditions. The search space was designed as shown in **Figure** **1**A, wherein the reduced ternary composition space (Li_9_PS_6.5_Cl‐Li_2.1_PS_3.39_Cl_0.31_‐Li_9_PS_5.39_Cl_3.23_), synthesis time (12, 24, and 36 h), temperature (500, 520, and 550 °C), and additives (either the inclusion or exclusion of carbon flakes) are schematically described. This type of multidimensional search space introduces an infinite number of possible decision variable sets (or vectors) when adopting mesh‐based full screening, leading to unviable experimental burdens. The reduced ternary composition space gradually increased as the PSO was iterated to later rounds; that is, the initial Li_9_PS_6.5_Cl‐Li_3.34_PS_3.95_Cl_0.43_‐Li_9_PS_5.85_Cl_2.3_ space was enlarged to Li_9_PS_6.5_Cl‐Li_2.1_PS_3.39_Cl_0.31_‐Li_9_PS_5.39_Cl_3.23_ space by the final (fifth) round. Note that the apex compositions in Figure 1A are labeled Li_2_S‐Li_2_P_5_‐LiCl for convenience. The instantaneous decision boundary alteration is common in PSO execution.^[^ ^53^ ^]^ The other processing variables (firing time, temperature, and carbon flakes) are discrete in nature, such that the variable can be compartmented while the PSO operates on the continuous variables.

The second (subsequent) PSO execution involved co‐doping with oxygen and three halides (Br, Cl, and I). A new composition search space was designed based on the first PSO execution result. The basic ternary composition search space (Li_6.14_PS_4.89_X_1.36_–Li_3.77_PS_3.93_X_0.9_– Li_6.15_PS_4.65_X_1.86_, X = halides) was significantly reduced relative to that of the first PSO execution. Instead, an additional LiCl‐LiBr‐LiI ternary composition search space was introduced along with the relative concentration of oxygen. Consequently, the composition search space included two ternary and one unary search spaces, as shown in Figure 1B. We constrained the firing time at 12 h and removed the carbon flakes –‐ consistent with the first PSO execution result. Only the firing temperature was retained as a processing variable in the second‐level PSO execution.

### 2.3 Stepwise Composition Evolution for Higher *σ*_ion_ via the PSO Implementation

Numerous studies have been conducted on argyrodite‐based solid‐state electrolytes, the composition of which deviates from the prototypical composition (Li_6_PS_5_Cl).^[^ ^18^, ^22^, ^33^, ^35^ ^]^ These studies have shown that the chemical constituents and their relative concentrations significantly influence the chemical environment for Li^+^ migration, which determine *σ*_ion_ values. However, a wide variation in *σ*_ion_ can also be attributed to both synthesis conditions and pellet/cell preparation methods. For example, high‐pressure pelletization followed by sintering typically result in a higher *σ*_ion_ (though, such methods are cost‐prohibitive at an industrial scale). The use of soft metallic blocking electrodes increased the measured *σ*_ion_. Different cell types may also contribute to the scatter in measured values. Therefore, fine‐tuning of the composition must be performed independently in a single laboratory while omitting steps that are unfavorable from an industrial point of view. It follows that the PSO‐driven stepwise approach is the most appropriate strategy because its characteristically rapid convergence enables a unit laboratory to carry out the optimization even in a multidimensional decision variable space.

Figure 1A illustrates the first PSO implementation, wherein all populations in each PSO round are represented. On the right side of every swarm, the objective function (*σ*_ion_) values of the individuals are also visualized. It is evident that the overall swarm distribution in the trigonal prism‐shaped search space moves downward (and forward) with repeated rounds. Both the average and maximum conductivities gradually improved as a function of the number of PSO rounds (Figure S1a, Supporting Information). The composition converged to Li_5.5_PS_4.5_Cl_1.5_, exhibiting the highest *σ*_ion_ of 3.89 mS cm^−1^ in the 5^th^ round. It should be noted that previous studies on the optimal composition in Li_7−_ *_x_* PS_6−_ *_x_* Cl*_x_* were inconsistent with the PSO‐nominated composition reported in this work. While the same optimal composition was reported by Adeli et al.,^[^ ^33^ ^]^ two other groups reported the highest *σ*_ion_ for Li_5.7_PS_4.7_Cl_1.3_ and Li_5.3_PS_4.3_Cl_1.7_.^[^ ^22^, ^35^ ^]^ The reported conductivities were also widely scattered in the range of 6.4–24 mS cm^−1^, indicating the sensitivity of the *σ*_ion_ measurements to synthetic, fabrication, and measurement conditions (as mentioned previously). The fact that the optimal composition obtained from the first PSO execution in the basic argyrodite composition search space did not correspond to a novel composition, but to a well‐known favorable composition, is noteworthy because it indicates the validity of the PSO‐based material discovery strategy. This composition, while not our final destination, is an important starting point for the ensuing co‐doping process.

The second PSO implementation involved additional co‐dopants such as O, Br, and I, in addition to the basic elements (Li, P, S, and Cl). The basic ternary compositional search space was designed around the best sample (Li_5.5_PS_4.5_Cl_1.5_) obtained from the first PSO execution and was downsized to Li_6.14_PS_4.89_X_1.36_–Li_3.77_PS_3.93_X_0.9_–Li_6.15_PS_4.65_X_1.86_ to avoid futile search efforts in the marginal region. Figure 1B presents a schematic of six swarms (six PSO rounds), while the rightmost side of every swarm (exhibiting *σ*_ion_ values for individuals in a given PSO round) presents the *σ*_ion_ improvement trend—indicating that both the average and maximum *σ*_ion_  for every PSO round followed an increasing trend (Figure S1b, Supporting Information). Figure 1B clearly shows the improvement in *σ*_ion_ as the PSO rounds proceeded. The highest *σ*_ion_ (7.78 mS cm^−1^) was obtained at a composition of Li_5.5_PS_4.5_Cl_0.89_Br_0.61_ in the 6^th^ round. Oxygen and iodine were completely eliminated; the fact that oxygen and iodine co‐doping exhibits no positive influence on *σ*_ion_ is well known.^[^ ^11^, ^14^, ^18^, ^44^, ^60^ ^]^ However, we observe that more sophisticated composition fine‐tuning has been achieved through a completely automated tandem PSO implementation.

The optimal argyrodite composition obtained from the tandem PSO executions (Li_5.5_PS_4.5_Cl_0.89_Br_0.61_) is comparable to that which emerged from initial PSO execution (Li_5.5_PS_4.5_Cl_1.5_), with the exception of the composition of mixed halides. Br incorporation significantly enhanced the *σ*_ion_. A number of studies have explored the effect of Br incorporation into Li_7−_ *_x_* PS_6−_ *_x_* Cl*_x_* ^[^ ^18^, ^32^, ^37^ ^]^—implying that the contribution of Br to *σ*_ion_ depends on the total halide concentration. For Li_6_PS_5_(Cl_1−_ *_x_* Br*_x_*), it has been claimed that the substitution of Cl^−^ by softer Br^−^ simultaneously decreases both the activation energy (*E*_a_) and the inter‐cage jump frequency (and thus, the prefactor *σ*_0_) for Li^+^ migration, which results in a decrease or marginal increase of *σ*_ion_ by Br‐doping.^[^ ^18^, ^32^ ^]^ This scenario, however, is unlikely to apply to argyrodite with high halide content. Recently, Patel et al. reported a gradual increase in *σ*_ion_ with Br‐doping up to *x* = 0.7 in Li_6−_ *_x_* PS_5−_ *_x_* ClBr*_x_*.^[^ ^37^ ^]^ This was shown to correspond to a continuous decrease in *E*_a_ and a slight increase in the jump rate with increased *x*, highlighting the importance of a flattened energy landscape for high *σ*_ion_. Our Li_5.5_PS_4.5_Cl_0.89_Br_0.61_, which has a high Br^−^/halide content, seems to follow the latter case, although the composition is not exactly identical (see below for *E*_a_ decrease).

The present investigation targeted the discovery of a novel, optimal composition with a high precision utilizing economical and viable procedures. In contrast to previous work on Li_6−_ *_x_* PS_5−_ *_x_* ClBr*_x_*,^[^ ^37^ ^]^ we allowed each constituent element to vary freely in the preset search space. The PSO‐obtained optimal composition was achieved through random, independent motion of each constituent element. Owing to this stochastic nature of PSO, we automatically reached Li_5.5_PS_4.5_Cl_0.89_Br_0.61_ without any human intervention, which is comparable (but not identical) to the known compositions in the literature.^[^ ^18^, ^32^, ^37^ ^]^ It should also be noted that the PSO‐optimized composition of the final compound (Li_5.5_PS_4.5_Cl_0.89_Br_0.61_) exhibited a *σ*_ion_ of 7.78 mS cm^−1^, which is less than the record‐high values which have previously been reported. However, we tested all such compositions suggested in the literature using the same synthesis, cell preparation, and characterization protocol—ultimately finding that our PSO‐based sample exhibits superior *σ*_ion_ relative to previously reported compositions. Time/energy‐intensive steps and procedures, which are unfeasible for industrial application, have been excluded from the synthesis/characterization protocol. Our industry‐friendly measurement protocol has also been shared with leading battery material/cell production companies such as Samsung SDI and Ecopro BM. Application of this reliable conductivity measurement protocol showed that the conductivity of Li_5.5_PS_4.5_Cl_0.89_Br_0.61_ was consistently higher than that reported for other compositions in the literature.

**Figure 2:**
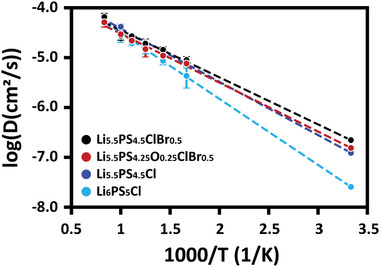
Diffusion coefficients and Arrhenius plots for argyrodites with the compositions closest to Li_6_PS_5_Cl, Li_5.5_PS_4.5_Cl_1.5_, Li_5.5_PS_4.5_Cl_0.89_Br_0.5_, and Li_5.5_PS_4.23_O_0.27_Cl_0.89_Br_0.61_. The error bar is the statistical uncertainty of each diffusivity data point.

We further validated the increase in *σ*_ion_ of Li_5.5_PS_4.5_Cl_0.89_Br_0.61_ relative to Li_5.5_PS_4.5_Cl_1.5_ via AIMD calculations. We prepared four input model structures: Li_6_PS_5_Cl, Li_5.5_PS_4.5_Cl_1.5_, Li_5.5_PS_4.5_ClBr_0.5_ (the closest possible model composition to Li_5.5_PS_4.5_Cl_0.89_Br_0.61_), and Li_5.5_PS_4.25_O_0.25_ClBr_0.5_ (the closest possible model composition to Li_5.5_PS_4.23_O_0.27_Cl_0.89_Br_0.61_). **Figure** **2** shows the AIMD‐calculated diffusivity as a function of temperature and the corresponding Arrhenius plots, which lead to the *σ*_ion_ and *E*_a_ listed in **Table** **1**. Although exact stoichiometry was not realized in the input model construction for AIMD, we approximated the real stoichiometry while ignoring the deviation. The calculated *σ*_ion_ were consistently higher than the experimental values owing either to interparticle resistance or structural defects in the real‐world examination. The varying trend was largely monotonic, an increase (or decrease) in the value of *σ*_ion_ (or *E*_a_) as we moved through Li_6_PS_5_Cl → Li_5.5_PS_4.5_Cl_1.5_ → Li_5.5_PS_4.5_ClBr_0.5_. As the oxygen incorporation deteriorated, however, the *σ*_ion_ (or *E*_a_) decreased (or increased) back to its level between Li_5.5_PS_4.5_Cl_1.5_ and Li_5.5_PS_4.5_ClBr_0.5_, which is also in good agreement with the experimental data. The details of the model construction and AIMD results are available in the Supporting Information (Figure S2).

**Table 1:** AIMD‐driven *σ*_ion_ and *E*_a_

Compound	*E*_a_ (eV)	*σ*_ion_ (mS cm^−1^)
Li_6_PS_5_Cl	0.263 ± 0.016	3.91
Li_5.5_PS_4.5_Cl_1.5_	0.212 ± 0.015	17.26
Li_5.5_PS_4.5_ClBr_0.5_	0.187 ± 0.024	30.57
Li_5.5_PS_4.25_O_0.25_ClBr_0.5_	0.195 ± 0.017	21.97

### 2.4 Oxygen Doping Effect

**Figure 3:**
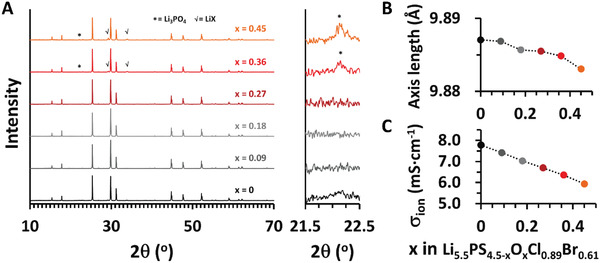
A) X‐ray diffraction patterns of Li_5.5_PS_4.5−_ *_x_* O*_x_* Cl_0.89_Br_0.61_ (*x* = 0, 0.09, 0.18, 0.27, 0.36, and 0.45). The right‐side panel shows the Li_3_PO_4_ impurity peaks for *x* > 0.27. B) Evolution of axis lengths with *x*, obtained from Le Bail refinement using the space group of *F*‐43*m*. The fitting profiles are also shown in Figure S3 (Supporting Information). C) Variation of *σ*_ion_ values of Li_5.5_PS_4.5−_ *_x_* O*_x_* Cl_0.89_Br_0.61_ with *x*. The *σ*_ion_ value for Li_5.5_PS_4.5−_ *_x_* O*_x_* Cl_0.89_Br_0.61_ samples in (C) along with other oxygen‐free argyrodites was listed up in Table S1 (Supporting Information).

Although oxygen was predictably eliminated over the course of PSO in order to maximize *σ*_ion_, we intentionally re‐introduced oxygen into the PSO‐nominated optimal composition of Li_5.5_PS_4.5_Cl_0.89_Br_0.61_ at the expense of ionic conductivity. This is necessary to enhance its commercial viability because, while argyrodite is known to be moisture‐sensitive and electrochemically unstable, oxygen doping is expected to resolve both these problems in one stroke.^[^ ^44^, ^60^, ^61^ ^]^ We prepared a series of argyrodite samples with various oxygen compositions (*x* = 0.09, 0.18, 0.27, 0.36, 0.45 in Li_5.5_PS_4.5−_ *_x_* O*_x_* Cl_0.89_Br_0.61_) and examined their structural stability by X‐ray diffraction (XRD). The XRD patterns shown in **Figure** **3**A clearly indicate the formation of impurity phases at *x* ≥ 0.36 (see the magnified view between 21.5° and 22.5°). The impurity phases were identified as Li_3_PO_4_ and LiX, which are commonly found in argyrodite when the oxygen content is high.^[^ ^60^ ^]^ An increase in *x* indicates the successful incorporation of oxygen into the lattice structure. Although not conspicuous, the (022) peaks at 2*θ* of ≈25.3° gradually moved toward a higher angle. Accordingly, Le Bail refinement revealed a continuous contraction of the unit cells from *a* = 9.8871 Å for *x* = 0 to *a* = 9.8831 Å for *x* = 0.45 (Figure 3B). The fitting profiles using the space group *F*‐43*m* are shown in Figure S3 (Supporting Information). As expected, *σ*_ion_ continuously decreased with increasing *x* (Figure 3C). The *σ*_ion_ values of 7.78, 7.42, 7.03, 6.70, 6.36, and 5.94 mS cm^−1^ were confirmed for *x* = 0, 0.09, 0.18, 0.27, 0.36, and 0.45, respectively.

**Figure 4:**
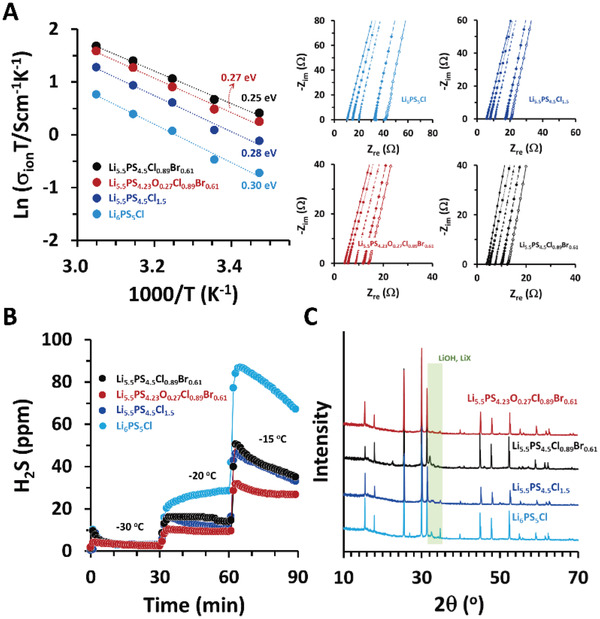
A) Arrhenius plots and corresponding EIS spectra for various argyrodites including the PSO‐obtained Li_5.5_PS_4.5_Cl_0.89_Br_0.61_ and oxygen‐tuned Li_5.5_PS_4.23_O_0.27_Cl_0.89_Br_0.61_. B) H_2_S gas release at dew points of −30 (RH = 1.5%), −20 (RH = 4.1%), and −15 °C (RH = 6.6%) in various argyrodites. C) Comparison between XRD patterns following exposure to a humid atmosphere (−15 °C, 30 min).

The direct correlation of *σ*_ion_ with *E*_a_ was also evident, implying that the change in the *E*_a_ modulated by the lattice softness plays a greater role in the ionic transport than the average vibrational frequencies of the lattice.^[^ ^18^ ^]^ Arrhenius plots and corresponding EIS spectra, shown in **Figure** **4**A, clearly demonstrate that the *E*_a_ decreased from 0.30 to 0.28 eV as Cl content increased from 1.0 to 1.5 (Li_6_PS_5_Cl → Li_5.5_PS_4.5_Cl_1.5_). Following partial substitution of Cl by Br (Li_5.5_PS_4.5_Cl_1.5_ → Li_5.5_PS_4.5_Cl_0.89_Br_0.61_), the *E*_a_ further decreased to 0.25 eV, which can be attributed to increased lattice softness. The incorporation of oxygen (Li_5.5_PS_4.5_Cl_0.89_Br_0.61_ → Li_5.5_PS_4.23_O_0.27_Cl_0.89_Br_0.61_), which is believed to aggravate lattice softness, resulted in an increase of *E*_a_ to 0.27 eV consistent with a decrease of *σ*_ion_.

Despite exhibiting a slight loss in *σ*_ion_, the moisture stability of Li_5.5_PS_4.23_O_0.27_Cl_0.89_Br_0.61_ was noticeably higher than that of oxygen‐free compounds. We monitored the amount of H_2_S released from the samples under atmospheres of various humidities (Figure 4B). Relative humidity (RH) was increased stepwise from 1.5% (dew point = −30 °C) to 4.1 and 6.6% (dew point = −20 and −15 °C, respectively); the change in ambient H_2_S concentrations was recorded over 30 min intervals at each RH. Except for initial spikes exhibited by the oxygen‐free compounds, no discernible difference in H_2_S concentrations for the various samples was observed at RH = 1.5% over the 30 min interval. Moisture stability, however, became more clearly distinguishable upon increasing the RH to 4.1 and 6.6%. The H_2_S release immediately increased following the increase in RH, with the highest H_2_S concentration observed for Li_6_PS_5_Cl. In contrast, the extent of this increase was noticeably less in Li_5.5_PS_4.5_Cl_1.5_ and Li_5.5_PS_4.5_Cl_0.89_Br_0.61_, which was not surprising given that hard‐acid Li^+^ prefers to bond to hard‐base H_2_O or OH^−^ rather than to softer S^2−^ or X^−^ bases.^[^ ^28^ ^]^ The partial replacement of soft‐base S^2−^ by hard‐base O^2−^ further mitigated the decomposition of argyrodite by moisture. The total amount of H_2_S released from Li_5.5_PS_4.23_O_0.27_Cl_0.89_Br_0.61_ within a given time regime (90 min) was approximately threefold lower than that from Li_6_PS_5_Cl. Accordingly, the XRD pattern of the moisture‐exposed Li_5.5_PS_4.23_O_0.27_Cl_0.89_Br_0.61_ is comparable to that of the pristine sample (Figure 4C). Only minor impurity peaks are observed between 31.5° and 35.5°. In contrast, the emergence of distinctive additional peaks (LiOH and LiX) was evident in the moisture‐exposed oxygen‐free compounds. Therefore, the oxygen‐doped Li_5.5_PS_4.23_O_0.27_Cl_0.89_Br_0.61_, which was derived from the PSO‐optimized Li_5.5_PS_4.5_Cl_0.89_Br_0.61_, appears to strike an appropriate balance between *σ*_ion_ and moisture resistance.

**Figure 5:**
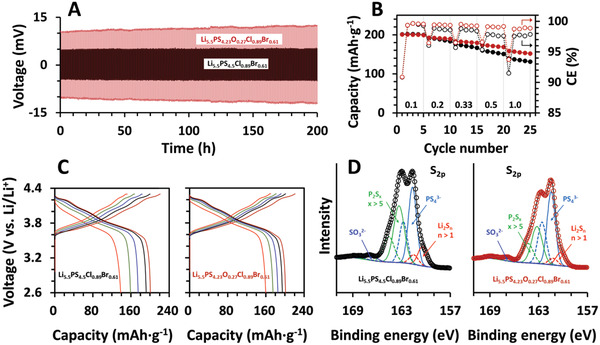
A) Voltage variation during Li plating/stripping at a current density of 1 mA cm^−2^ (0.5 mAh cm^−2^) in Li ‖ Li symmetric cells. B) Discharge capacities and CE changes. C) Representative C/D profiles in Li ‖ Li(Ni_0.9_Co_0.05_Mn_0.05_)O_2_ cells at various C‐rates. Li(Ni_0.9_Co_0.05_Mn_0.05_)O_2_ with no protective coating was used to compare the interfacial stability more clearly. D) High‐resolution XPS spectra for sulfur 2p after C/D cycles. The presence of SO_3_^2−^ is likely due to a brief exposure of the specimens to air during transfer.

We also examined the electrochemical stability of Li_5.5_PS_4.23_O_0.27_Cl_0.89_Br_0.61_ in contact with Li. The improvement of interfacial stability has already been reported in a handful of oxygen‐containing argyrodites such as (Li_5.7_Zn_0.15_)P(S_4.85_O_0.15_)Br,^[^ ^44^ ^]^ Li_6_P(S_4.7_O_0.3_)Br,^[^ ^60^ ^]^ and Li_6.3_(P_0.7_Sn_0.3_)(S_4.4_O_0.6_)I.^[^ ^61^ ^]^ These studies have reported a decrease in overpotential and suppression of dendrites during Li plating/stripping in Li symmetric cells implemented with oxide‐doped argyrodites. However, our Li_5.5_PS_4.23_O_0.27_Cl_0.89_Br_0.61_ has demonstrated the opposite behavior (**Figure** **5**A). When cycled at 1 mA cm^−2^ (0.5 mAh cm^−2^), Li_5.5_PS_4.23_O_0.27_Cl_0.89_Br_0.61_ revealed a slightly higher overpotential (≈±10 mV) than Li_5.5_PS_4.5_Cl_0.89_Br_0.61_ (≈±5 mV). No sign of a dendrite‐induced short‐circuit was observed for either compound during 200 plating/stripping cycles. The stability of oxygen‐free Li_5.5_PS_4.5_Cl_0.89_Br_0.61_ was particularly notable given that previous studies have reported an immediate cell failure with oxygen‐free argyrodites.^[^ ^44^, ^60^, ^61^ ^]^ For example, Zhang et al. showed that a symmetric cell with Li_6_PS_5_Br experienced a short‐circuit after 10 cycles even at relatively low current densities (< 0.5 mA cm^−2^),^[^ ^60^ ^]^ contrasting with our result (>100 cycles at 1.0 mA cm^−2^). This discrepancy implies that either the greater halogen content induces a higher interfacial stability or that Cl^−^ ions promote interfacial stability more effectively than Br^−^ ions. Hence, the intrinsic stability of Li_5.5_PS_4.5_Cl_0.89_Br_0.61_ seems to render the effect of oxygen‐doping indiscernible. In fact, if the effect of the lower *σ*_ion_ of Li_5.5_PS_4.23_O_0.27_Cl_0.89_Br_0.61_ is wholly reflected in the overpotentials, plating/stripping should occur at ±25 mV in Li_5.5_PS_4.23_O_0.27_Cl_0.89_Br_0.61_.

To confirm the influence of oxygen‐doped argyrodite on the full cell performance, we constructed cells with a configuration of Li ‖ SSE ‖ Li(Ni_0.9_Co_0.05_Mn_0.05_)O_2_ and compared the charge/discharge (C/D) behaviors of the cells implemented with Li_5.5_PS_4.23_O_0.27_Cl_0.89_Br_0.61_ and Li_5.5_PS_4.5_Cl_0.89_Br_0.61_. Note that we intentionally used Li(Ni_0.9_Co_0.05_Mn_0.05_)O_2_, which was not surface‐modified, to more clearly observe the effect of oxygen doping at the cathode–SSE interface. Figure 5B shows the variation in the discharge capacity and columbic efficiency (CE) at various C‐rates (1C = 200 mA g^−1^). It can be observed that, while the difference in discharge capacities is negligible at 0.1C, the capacity of Li_5.5_PS_4.23_O_0.27_Cl_0.89_Br_0.61_ gradually increased as the C‐rate increased. For example, the capacity of ≈154 mAh g^−1^ was 14% higher than that of ≈135 mAh g^−1^. This superior capacity observed at high C‐rates correlates with the higher CE of Li_5.5_PS_4.23_O_0.27_Cl_0.89_Br_0.61_. As with the capacity difference, the higher CE of Li_5.5_PS_4.23_O_0.27_Cl_0.89_Br_0.61_ becomes more distinct as the C‐rate increases, which is believed to be due to the improved interfacial stability at Li_5.5_PS_4.23_O_0.27_Cl_0.89_Br_0.61_/Li(Ni_0.9_Co_0.05_Mn_0.05_)O_2_ under high‐voltage charge (we applied a constant voltage of 4.3 V for 30 min at the end of the charge). The corresponding C/D profiles at various C rates are shown in Figure 5C.

Following the C/D cycles, the cells were disassembled and the chemical state of sulfur in the SSE in contact with the cathode was examined using X‐ray photoelectron microscopy (XPS). Because sulfide in argyrodites is known to be oxidized to lithium polysulfides (Li_2_S*_n_*, *n* > 1) or phosphorus polysulfides (P_2_S*_x_*, *x* > 5) at high voltages,^[^ ^62^, ^63^ ^]^ we compared the XPS spectra for S_2p_ to examine the difference in oxidative stability at the cathode interface (Figure 5D). The XPS spectra of the two compounds (Li_5.5_PS_4.5_Cl_0.89_Br_0.61_ and Li_5.5_PS_4.23_O_0.27_Cl_0.89_Br_0.61_) demonstrated a discernible difference in intensity, especially at high binding energies (BEs). Relative to the peaks at ≈162 eV (light blue lines), the peak intensities at ≈164 eV (green lines) were significantly less for Li_5.5_PS_4.23_O_0.27_Cl_0.89_Br_0.61_, indicating a lower degree of P_2_S*_x_* formation (96% in Li_5.5_PS_4.5_Cl_0.89_Br_0.61_ vs 64% in Li_5.5_PS_4.23_O_0.27_Cl_0.89_Br_0.61_ for S_2p3/2_ peaks). The similar trend was observed in the low‐BE region (i.e., less Li_2_S*_n_* peak intensity in Li_5.5_PS_4.23_O_0.27_Cl_0.89_Br_0.61_). The improved interfacial stability owing to the incorporation of oxygen is believed to contribute to the higher capacity of Li_5.5_PS_4.23_O_0.27_Cl_0.89_Br_0.61_, which is more conspicuous at high C‐rates.

### 2.5 Structural Features of Li_5.5_PS_4.23_O_0.27_Cl_0.89_Br_0.61_

**Figure 6:**
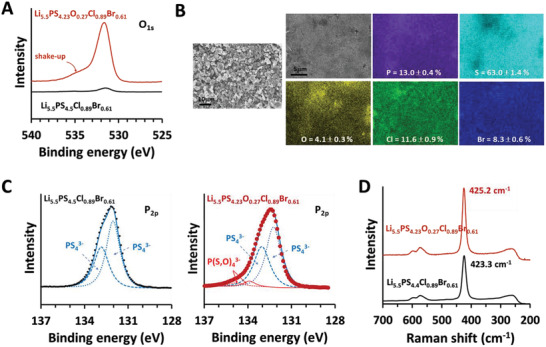
A) High‐resolution XPS spectra of O_1s_ for Li_5.5_PS_4.5_Cl_0.89_Br_0.61_ and Li_5.5_PS_4.23_O_0.27_Cl_0.89_Br_0.61_. B) FESEM image and corresponding EDX maps of Li_5.5_PS_4.23_O_0.27_Cl_0.89_Br_0.61_. C) High‐resolution XPS spectra of P_2p_ and D) Raman spectra for Li_5.5_PS_4.5_Cl_0.89_Br_0.61_ and Li_5.5_PS_4.23_O_0.27_Cl_0.89_Br_0.61_.

Prior to characterizing the microstructural features of Li_5.5_PS_4.23_O_0.27_Cl_0.89_Br_0.61_, we confirmed the nominal compositions. The comparison of the oxygen concentrations, examined by high‐resolution XPS, clearly revealed the existence of a high level of oxygen in Li_5.5_PS_4.23_O_0.27_Cl_0.89_Br_0.61_ relative to its negligible amount in Li_5.5_PS_4.5_Cl_0.89_Br_0.61_ (**Figure** **6**A). The peak shape was also Gaussian‐symmetric along with a shake‐up satellite peak, implying a single chemical state of oxygen. A trace amount of oxygen in Li_5.5_PS_4.5_Cl_0.89_Br_0.61_ appeared due to contamination in air during sample transfer for XPS measurements.

The relative ratios of all elements (except Li) were determined using energy‐dispersive X‐ray spectroscopy (EDX). Figure 6B shows field‐emission scanning electron microscopy (FESEM) images and corresponding EDX maps of Li_5.5_PS_4.23_O_0.27_Cl_0.89_Br_0.61_. Because the as‐prepared powder was ground via planetary ball milling, the particles exhibited an irregular morphology with a diameter of 5 *µ*m. The relative compositions of P/S/O/Cl/Br were obtained for a pressed pellet, which exhibited a smooth surface with few pinholes. The relative compositions examined for more than 10 samples are indicated in each EDX map—corresponding to PS_4.8_O_0.30_Cl_0.89_Br_0.64_. Given the inherent uncertainty in quantifying compositions using EDX (≈±5–10%), the EDX‐calculated composition suggested a correspondence between the actual and the nominal compositions at an acceptable level.

As mentioned above, there have been a number of studies on oxygen‐doped argyrodites;^[^ ^44^, ^60^, ^61^ ^]^ however, the crystallographic position of oxygen has not yet been clearly specified. Zhang et al. reported that oxygen atoms do not substitute sulfur atoms at the 16e site in Li_6_P(S_4.7_O_0.3_)Br_1.0_, resulting in no change in the XRD patterns and Raman spectra following oxygen doping.^[^ ^60^ ^]^ However, this was not the case with Li_5.5_PS_4.23_O_0.27_Cl_0.89_Br_0.61_. The high‐resolution XPS spectra of P_2p_ showed a discernible tail on the high‐BE side in the oxygen‐doped sample (Figure 6C). Although not conspicuous, the fitting quality became acceptable (*χ* ^2^ = 4.5) only when small peaks were included at ≈134 and 135 eV. Furthermore, the area of small peaks relative to that of main peaks was ≈6.8%, which was comparable to the value in P(S_3.73_O_0.27_)^3−^. Hence, oxygen atoms most likely substituted the sulfur atoms of PS_4_^3−^ in Li_5.5_PS_4.23_O_0.27_Cl_0.89_Br_0.61_.

The substitution of sulfur PS_4_ with oxygen was further validated by comparing the Raman spectra of Li_5.5_PS_4.5_Cl_0.89_Br_0.61_ and Li_5.5_PS_4.23_O_0.27_Cl_0.89_Br_0.61_ (Figure 6D). Raman spectra of both samples showed a series of peaks corresponding to the vibrational modes of PS_4_ tetrahedra (230–310, 400–450, and 530–620 cm^−1^).^[^ ^60^ ^]^ If oxygen is substituted for sulfur in PS_4_ tetrahedra, the short P‐O bonds can induce a blue shift in the Raman bands; we closely compared the peak positions. As expected, the most intense peak was observed at 423.3 cm^−1^ for Li_5.5_PS_4.5_Cl_0.89_Br_0.61_, which is attributed to A_1_ symmetric stretching vibration of PS_4_ units; this is shifted to 425.2 cm^−1^ in Li_5.5_PS_4.23_O_0.27_Cl_0.89_Br_0.61_, indicating the location of oxygen in 16e sites.

**Figure 7:**
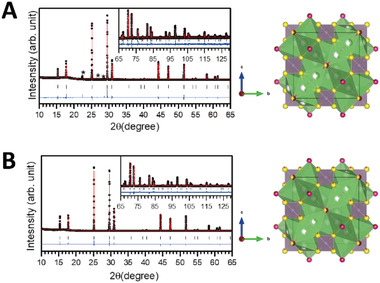
Rietveld refinement fit on synchrotron XRD data for A) Li_5.5_P S_4.5_Cl_0.89_Br_0.61_ and B) Li_5.5_PS_4.23_O_0.27_Cl_0.89_Br_0.61_ adopting a cubic structure with an $F\bar{4}3m$ space group in the 2*θ*‐range (10°–130.5°) and a step‐size of 0.005°. Black dots, red lines, and blue lines represent observed, calculated, and difference profiles, respectively. The vertical tick marks above the difference profile denote the positions of Bragg reflections. A very small fraction of an unidentified impurity marked with an asterisk (*) is also present in the XRD pattern. The schematics for both the refined structures are also presented.

**Figure** **7** presents the synchrotron light source XRD pattern for Li_5.5_P S_4.5_Cl_0.89_Br_0.61_ and Li_5.5_PS_4.23_O_0.27_Cl_0.89_Br_0.61_; the Rietveld refinement result is listed in Table S1 (Supporting Information). Rietveld refinements on the synchrotron XRD data were carried out using Fullprof^[^ ^64^ ^]^ while considering the structure of Li_6_PS_5_X (X = Cl, Br, I)^[^ ^18^ ^]^ as an initial model, exhibiting a cubic structure with a $F\bar{4}3m$ space group. In the refinement, the profile shape and background were modeled using a pseudo‐Voigt function and a linear interpolation between the set background, respectively. It was also necessary to use anisotropic peak broadening in the case of synchrotron XRD data. Refinement parameters such as scale factor, background, half‐width parameters, lattice parameters, positional coordinates, and thermal parameters, were varied in the course of refinement. Occupancy parameters at respective sites were fixed according to stoichiometric composition for Li_5.5_PCl_0.89_Br_0.61_S_4.5_ and Li_5.5_PS_4.23_O_0.27_Cl_0.89_Br_0.61_. All the ions (Cl, Br, and S) occupied the *4a* site while the 4d site was occupied only by Cl and Br ions; placing the S ion on *4d* site did not result in a good fit. All the oxygen atoms substituted sulfur atoms at the 16e site. It can be observed from Figure 7A,B that a very good fit between the observed and calculated profiles was obtained with an almost flat difference profile, along with favorable values for the corresponding agreement factors (*R*_p_, *R*_wp_, *R*_exp_, and *χ* ^2^). The pristine argyrodite structure was maintained without having been impacted by the incorporation of O and Br. The results of Rietveld refinement are presented in Table S2 (Supporting Information)

## 3 Conclusion

The experimental‐data‐driven two‐step PSO, titled the tandem PSO algorithm, was developed and implemented in this study. While a single‐step PSO would have resulted in a huge decision variable space (=search space), the tandem PSO algorithm enabled us to significantly save experimental expenditures by reducing the search space. Using this approach, we discovered three promising argyrodite compositions: Li_5.5_PS_4.5_Cl_1.5_, Li_5.5_PS_4.5_Cl_0.89_Br_0.61_, and Li_5.5_PS_4.5−_ *_x_* O*_x_* Cl_0.89_Br_0.61_.

Through implementation of the tandem PSO algorithm, we discovered the optimal composition of lithium argyrodite with the highest *σ*_ion_ in a multidimensional search space. First, the PSO execution was performed within typical composition ranges and by varying certain processing conditions. The composition converged to Li_5.5_PS_4.5_Cl_1.5_ in the fifth round, which corresponded to the highest *σ*_ion_ of 3.89 mS cm^−1^. Next, the composition of Li_5.5_PS_4.5_Cl_1.5_ was finely tuned in the vicinity of the composition. During the second PSO execution, we introduced co‐dopants such as O, Br, and I and reached the optimal composition of Li_5.5_PS_4.5_Cl_0.89_Br_0.61_ in the 6^th^ round, which exhibited the highest *σ*_ion_ value of 7.78 mS cm^−1^. Throughout the process (synthesis of argyrodites and measurement of *σ*_ion_), we used neither excessive milling/densification nor complicated sintering protocols. We only adopted industrially feasible processes (e.g., a conventional cold‐pressing procedure for pellet preparation).

To the best composition identified from tandem PSO (Li_5.5_PS_4.5_Cl_0.89_Br_0.61_), we intentionally reintroduced oxygen to mitigate the inherent challenges of both moisture susceptibility and electrochemical instability (Li_5.5_PS_4.5−_ *_x_* O*_x_* Cl_0.89_Br_0.61_). The gradual addition of O resulted in a slight compromise in *σ*_ion_ (6.70 mS cm^−1^) and *E*_a_ (0.27 eV) in a phase pure Li_5.5_PS_4.23_O_0.27_Cl_0.89_Br_0.61_. However, the slight degradation after oxygen incorporation was fully compensated by the noticeable improvement in environmental stability (the least degree of H_2_S release and negligible changes in the structure in a humid atmosphere). Li_5.5_PS_4.23_O_0.27_Cl_0.89_Br_0.61_ also improved the interfacial stability when in contact with the Li(Ni_0.9_Co_0.05_Mn_0.05_)O_2_ cathode, which contributed to a greater specific capacity during fast charge/discharge. The structural and chemical features of Li_5.5_PS_4.23_O_0.27_Cl_0.89_Br_0.61_ argyrodites were comprehensively characterized using synchrotron X‐ray diffraction, Raman spectroscopy, and X‐ray photoelectron spectroscopy.

## 4 Experimental Section

### Sample Preparation and Characterization

Argyrodite compounds during the course of PSO were synthesized with appropriate amounts of Li_2_S (Alfa Aesar, 99.9%), Li_2_O (Alfa Aesar, 99.5%), P_2_S_5_ (Sigma‐Aldrich, 99%), LiCl (Sigma‐Aldrich, 99%), LiBr (Sigma‐Aldrich, 99%), and LiI (Sigma‐Aldrich, 99%). The powders were hand‐ground for 30 min, sealed in quartz tubes under vacuum, and subjected to heat treatment at preset temperatures for 12–36 h (heating rate = +3 °C min^−1^, cooling rate = −2 °C min^−1^). All the processes were performed inside a glove box (O_2_ and H_2_O less than 0.1 ppm).

X‐ray diffraction patterns of the as‐synthesized samples were recorded using a Cu‐K*α* source (Ultima IV, Rigaku Corp.). During the measurements, the sample was protected from oxygen and moisture through the use of a purpose‐built sample holder. A synchrotron X‐ray source at the Pohang Accelerator Laboratory (beamline 3D, Korea) was used to collect data on a MAR345 image plate with an incident wavelength of 1.0332 Å (12 keV). For Raman analysis, the sample was placed under an airtight seal in a specially fabricated glass slide. Raman spectra were collected on a Jasco NRS‐2100 laser Raman spectrometer (532 nm laser line). A survey scan from 200 to 700 cm^−1^ using 50 accumulations (10 s per accumulation) was collected to observe any changes in the PS_4_^3−^ peak positions due to oxide doping. XPS spectra were obtained using a Thermo Fisher Scientific (K‐Alpha) electron spectrometer with an Al‐K*α* X‐ray source (excitation energy = 1486.6 eV). FESEM studies were performed using a JEOL JSM‐7610F Plus instrument equipped with an EDX spectroscope. The concentration of H_2_S gas generated via the reaction between the argyrodite and moisture was monitored in real time. N_2_ gas with controlled humidity (PPMG101, Roscid Technologies) was continuously injected into a purpose‐built sample holder and H_2_S concentration was detected using an air quality monitoring sensor (aeroqual 500 series, Visitech Co., Korea).

### Electrochemical Test

The as‐prepared powder (200 mg) was thoroughly ground and placed in a polyoxymethylene (POM) mold (13 mm in diameter). The powder was then pelletized between indium foils (50 *µ*m thick) at 370 MPa. The typical pellet thickness was ≈800 *µ*m, which corresponds to ≈95% (1.78 g cm^−3^) of the theoretical density (1.86 g cm^−3^ for Li_6_PS_5_Cl,). Electrochemical impedance spectra (EIS) were recorded by applying a sine wave with an amplitude of ±10.0 mV at frequencies ranging from 1 MHz to 0.01 Hz (SP2, WonATech). To obtain the temperature‐dependent EIS spectrum, the cell was placed in an N_2_‐filled oven and rested for 1.5 h to reach thermal equilibrium at each temperature.

For the symmetric cell tests, the argyrodite powder was pelletized first at 370 MPa and then sandwiched between two Li foils under mild pressure (≈10 kPa). For the solid‐state cell tests, the cathode composite was prepared from a mixture of Li(Ni_0.9_Co_0.05_Mn_0.05_)O_2_ (50 wt%), SSE (40 wt%, extensively ground to an average diameter of <1 *µ*m), and carbon fiber (10 wt%). The cathode mixture (20 mg) was placed on an already pelletized SSE (200 mg) and pressurized with a Li foil at the bottom.

### AIMD Calculation

To examine the Li‐ion conductivity and activation energies of the selected candidates, AIMD^[^ ^65^, ^66^ ^]^ calculations were implemented at 600, 700, 800, 900, 1000, and 1200 K. The AIMD simulation lasted 250 ps with a time step of 2 fs and was based on the canonical ensemble (NVT) and Nose´–Hoover thermostat algorithm. The calculation protocol proposed by Fang and Jena^[^ ^39^ ^]^ and He et al.^[^ ^67^ ^]^ was followed. The number of possible configurations for an input model structure was 10^15^, which was intractable from a practical perspective. It is conventional to select entries with relatively low Coulomb energy from as many random configurations as possible.^[^ ^29^, ^37^, ^38^ ^]^ However, a genetic algorithm (GA) was introduced to pinpoint entries with relatively low Coulomb energy in a more systematic manner.

Figure S2A,B (Supporting Information) presented the result of the GA implementation, exhibiting an evolution trend (i.e., a decreasing trend in the Coulomb energy). Each generation consisted of 100 configurations; the Coulomb energy value for every generation was represented as a violin plot. The ten lowest‐Coulomb‐energy entries were selected for Li_6_PS_5_Cl and Li_5.5_PS_4.5_Cl_1.5_; the corresponding DFT‐calculated formation enthalpies were plotted in Figure S2C (Supporting Information). For Li_5.5_PS_4.5_ClBr_0.5_ and Li_5.5_PS_4.25_O_0.25_ClBr_0.5_, additional compositional configurations were created by substitution with identical valence state ions (e.g., Br→Cl and O→S substitutions); the DFT‐calculated formation enthalpies of these additional configurations are shown in Figure S2D (Supporting Information). This GA‐driven systematic configurational treatment for the AIMD calculation was shown to be demonstrably accurate and computationally inexpensive.

### Statistical Analysis

Every ionic conductivity value represents an average of three argyrodite samples. When an extremely deviant outlier was detected among the three argyrodite samples, i.e., when the maximum difference between the three samples exceeded 10%, two more samples were synthesized and two extremely deviant samples were removed out of a total of five. The resultant three samples were used for averaging. Consequently, the maximum difference between the three samples never exceeded 10%.

## Conflict of Interest

The authors declare no conflict of interest.

## Supporting information

Supporting Information

## Data Availability

The data that support the findings of this study are available from the corresponding author upon reasonable request.
