# Supplementary material for: Optimal Composition of Li Argyrodite with Harmonious Conductivity and Chemical/Electrochemical Stability: Fine‐Tuned Via Tandem Particle Swarm Optimization
Source: Adv Sci (Weinh). 2022 Jul 21;9(28):2201648. doi: 10.1002/advs.202201648 (PMC9534954; doi:10.1002/advs.202201648)
Supplement: Supplementary file 1 — Supporting Information [file ADVS-9-2201648-s001.pdf]

Optimal composition of Li argyrodite with harmonious conductivity and chemical/electrochemical stability: fine-tuned via tandem particle swarm optimization

Sunggeun Shim, Woon Bae Park, Jungmin Han, Jinhyeok Lee, Byung Do Lee, Jin-Woong Lee, Jung Yong Seo, S. J. Richard Prabakar, Su Cheol Han, Satendra Pal Singh, Chan-Cuk Hwang, Docheon Ahn, Sangil Han, Kyusung Park, Kee-Sun Sohn, and Myoungho Pyo**

S. Shim,[†] J. Lee, B. D. Lee, J.-W. Lee, S. P. Singh, K.-S. Sohn
Faculty of Nanotechnology and Advanced Materials Engineering,
Sejong University,
Seoul 05006, Republic of Korea.
E-mail: kssohn@sejong.ac.kr

W. B. Park,[†] J. Y. Seo, S. J. R. Prabakar, S. C. Han, M. Pyo
Department of Advanced Components and Materials Engineering,
Sunchon National University,
Chonnam 57922, Republic of Korea.
E-mail: mho@sunchon.ac.kr

C.-C. Hwang, D. Ahn
Beamline Department,
Pohang Accelerator Laboratory,
Pohang 790-784, Republic of Korea.

J. Han, S. Han, K. Park
Next Generation Development Team,
Samsung SDI R&D Center,
Suwon-si, Gyeonggi-do, 16678, Republic of Korea.

[†] These authors contributed equally to this work.

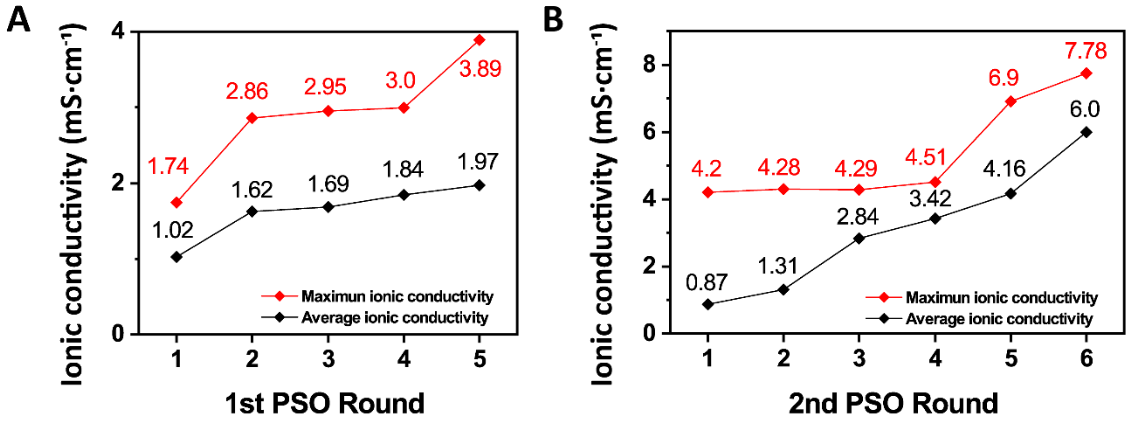

Figure S1. Maximum and average σ_{ion} as a function of swarm number (PSO round number) for the (A) first and (B) second PSO executions.

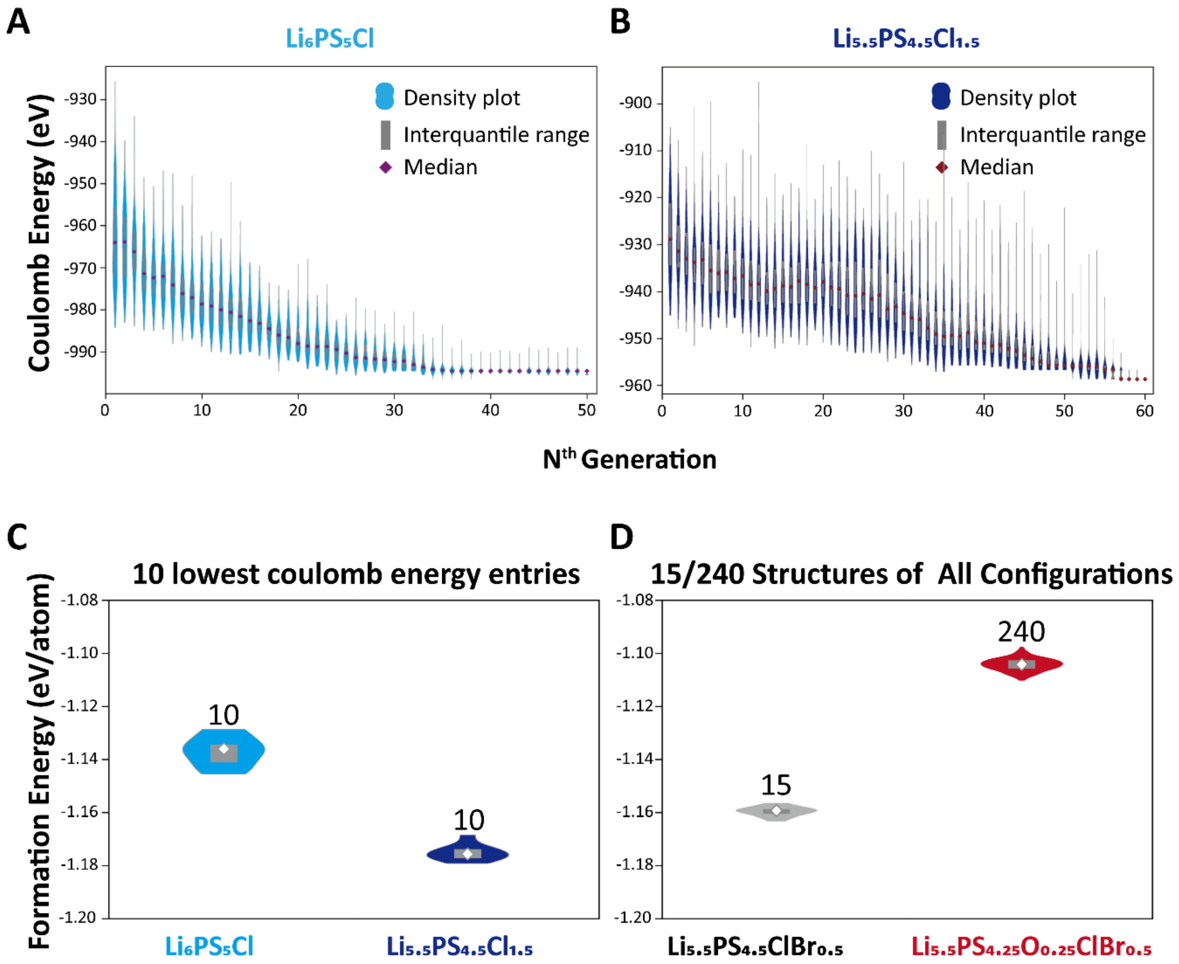

Figure S2. GA implementation and the resultant configurational selection. (A) GA iteration result up to the 50th generation for Li_6PS_5Cl, and (B) GA iteration result up to the 60th generation for $Li_{5.5}PS_{4.5}Cl_{1.5}$. (C) DFT-calculated formation enthalpy for 10 lowest Coulomb energy entries for Li_6PS_5Cl and $Li_{5.5}PS_{4.5}Cl_{1.5}$. (D) DFT-calculated formation enthalpy for the additional compositional configurations at the lowest Coulomb energy for $Li_{5.5}PS_{4.5}ClBr_{0.5}$ and $Li_{5.5}PS_{4.25}O_{0.25}ClBr_{0.5}$.

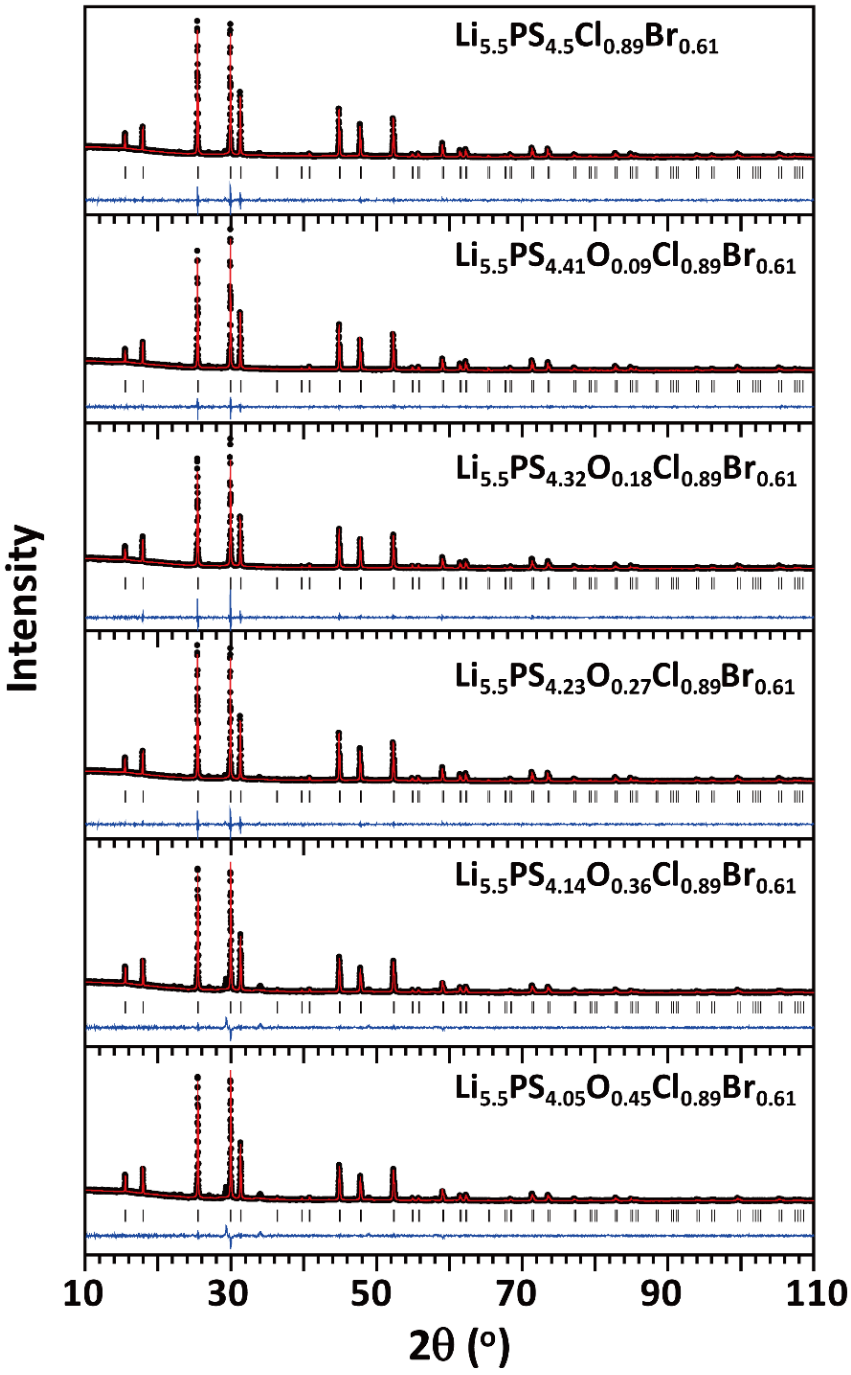

Figure S3. Full-pattern Le Bail refinement of $Li_{5.5}PS_{4.5-x}O_xCl_{0.89}Br_{0.61}$ (x = 0, 0.09, 0.18, 0.27, 0.36, and 0.45) using a cubic structure in the $F\bar{4}3m$ space group. The black dots, red line, and blue line represent observed, calculated, and difference profiles, respectively. The vertical tick marks above the difference profile denote the positions of Bragg reflections.

Table S1. The ion conductivity (σ^{ion}) for $Li_{5.5}PS_{4.5-x}O_xCl_{1.5}$ samples in Figure 3C along with those for other oxygen-free argyrodite samples.

Compound	σ_{ion} (mS·cm^{-1})
Li_6PS_5Cl	**1.98**
$Li_{5.5}PS_{4.5}Cl_{1.5}$	**3.89**
$Li_{5.5}PS_{4.5}Cl_{0.89}Br_{0.61}$	**7.78**
$Li_{5.5}PS_{4.41}O_{0.09}Cl_{0.89}Br_{0.61}$	**7.42**
$Li_{5.5}PS_{4.32}O_{0.18}Cl_{0.89}Br_{0.61}$	**7.03**
$Li_{5.5}PS_{4.23}O_{0.27}Cl_{0.89}Br_{0.61}$	**6.70**
$Li_{5.5}PS_{4.14}O_{0.36}Cl_{0.89}Br_{0.61}$	**6.36**
$Li_{5.5}PS_{4.05}O_{0.45}Cl_{0.89}Br_{0.61}$	**5.94**

Table S2. Atomic coordinates, atomic displacement parameter (U) and site occupancy factor (SOF) obtained after the Rietveld refinement on synchrotron powder diffraction data of (A) $Li_{5.5}P\ Cl_{0.89}Br_{0.61}S_{4.5}$ and (B) $Li_{5.5}PCl_{0.89}Br_{0.61}S_{4.23}O_{0.27}$.

A

Atom	Wyckoff site	x	y	z	U (Å^2)	SOF
Li	*48h*	0.3395 (6)	-0.0444 (8)	0.6606 (6)	0.024	0.458
P	*4b*	0.00000	0.00000	0.50000	0.0249 (6)	1.0
Cl1	*4a*	0.00000	0.00000	0.00000	0.0254 (5)	0.098
Cl2	*4d*	0.25000	0.25000	0.75000	0.0227 (5)	0.791
Br1	*4a*	0.00000	0.00000	0.00000	0.0254 (5)	0.401
Br2	*4d*	0.25000	0.25000	0.75000	0.0227 (4)	0.209
S1	*4a*	0.00000	0.00000	0.00000	0.0254 (5)	0.501
S2	*16e*	0.11884 (9)	-0.11884 (9)	0.61884 (9)	0.0413 (4)	1.0

Crystallographic Data

Source : Synchrotron
Chemical formula : $Li_{5.5}P\ Cl_{0.89}Br_{0.61}S_{4.5}$
Formula weight : 293.737
Temperature : 295K
Pressure (if not ambient): Atmospheric
Wavelength : 1.5212Å
Crystal system : Cubic
Space group (No.) : $F\bar{4}3m$ (216)
a, b, c, α, β, γ : a = b = c = 9.88599 (1) Å ; α = β = γ = 90°
V (Å^3) :966.184(3)
Z : 4
d-space range : 5.7077- 0.8385
R factors : R_p = 7.61, R_{wp} = 10.5, R_{exp}= 8.20 and χ^2 = 1.63.

B

Atom	Wyckoff site	x	y	z	U (Å^2)	SOF
Li	*48h*	0.3187 (5)	-0.0349 (7)	0.6814 (5)	0.04530	0.458
P	*4b*	0.00000	0.00000	0.50000	0.0283 (4)	1.0
Cl1	*4a*	0.00000	0.00000	0.00000	0.0412 (3)	0.098
Cl2	*4d*	0.25000	0.25000	0.75000	0.0374 (3)	0.791
Br1	*4a*	0.00000	0.00000	0.00000	0.0412 (3)	0.401
Br2	*4d*	0.25000	0.25000	0.75000	0.0374 (3)	0.209
S1	*4a*	0.00000	0.00000	0.00000	0.0412 (3)	0.501
S2	*16e*	0.11830 (6)	-0.11830 (6)	0.61830 (6)	0.0453 (3)	0.932
O	*16e*	0.11830 (6)	-0.11830 (6)	0.61830 (6)	0.0453 (3)	0.068

Crystallographic Data

Source : Synchrotron
Chemical formula : $Li_{5.5}PCl_{0.89}Br_{0.61}S_{4.23}O_{0.27}$
Formula weight : 289.397
Temperature : 295K
Pressure (if not ambient): Atmospheric
Wavelength : 1.5221Å
Crystal system : Cubic
Space group (No.) : $F\bar{4}3m$ (216)
a, b, c, α, β, γ : a = b = c = 9.88088 (1) Å ; α = β = γ = 90°
V (Å^3) : 964.688 (1)
Z : 4
d-space range : 5.7077- 0.8385
R factors : R_p = 6.96, R_{wp} = 9.68, R_{exp}= 6.84 and χ^2 = 2.00.

Table S3. The decision variable (composition, firing temperature, carbon paper) and objective function (Li ionic conductivity) values for entire generation (swarms) of the tandem PSO iteration.

Sample No.	Composition	Temperature (℃)	Time (h)	Carbon paper	Ionic conductivity (mS・cm^{-1})
1_1-1	$Li_{6.837}PS_{5.289}Cl_{1.258}$	500	36	x	**1.16**
1_1-2	$Li_{8.59}PS_{5.803}Cl_{1.984}$	500	36	x	**0.69**
1_1-3	$Li_{8.425}PS_{5.868}Cl_{1.69}$	500	24	x	**0.69**
1_1-4	$Li_{5.024}PS_{4.582}Cl_{0.86}$	550	24	x	**0.89**
1_1-5	$Li_{8.112}PS_{5.991}Cl_{1.13}$	520	24	o	**0.79**
1_1-6	$Li_{8.266}PS_{5.93}Cl_{1.405}$	520	24	x	**0.77**
1_1-7	$Li_{6.288}PS_{4.991}Cl_{1.307}$	520	24	x	**1.64**
1_1-8	$Li_{3.662}PS_{4.063}Cl_{0.537}$	520	12	o	**0.05**
1_1-9	$Li_{6.288}PS_{4.991}Cl_{1.307}$	520	12	x	**1.74**
1_1-10	$Li_{7.22}PS_{5.373}Cl_{1.474}$	550	24	o	**1.32**
1_1-11	$Li_{7.345}PS_{5.309}Cl_{1.727}$	550	36	x	**1.28**
1_1-12	$Li_{7.253}PS_{5.652}Cl_{0.95}$	500	24	o	**0.79**
1_1-13	$Li_{4.636}PS_{4.441}Cl_{0.754}$	520	12	o	**0.98**
1_1-14	$Li_{5.567}PS_{4.906}Cl_{0.756}$	500	12	o	**1.03**
1_1-15	$Li_{5.647}PS_{4.849}Cl_{0.949}$	500	24	x	**0.91**
1_1-16	$Li_{6.413}PS_{5.178}Cl_{1.058}$	520	24	o	**1.6**
1_1-17	$Li_{6.668}PS_{5.187}Cl_{1.294}$	550	36	x	**0.9**
1_1-18	$Li_{6.777}PS_{5.125}Cl_{1.527}$	520	24	x	**1.19**

Sample No.	Composition	Temperature (℃)	Time (h)	Carbon paper	Ionic conductivity (mS · cm-1)
1_2-1	$Li_{6.777}PS_{5.199}Cl_{1.38}$	500	24	o	**2.09**
1_2-2	$Li_{7.921}PS_{5.557}Cl_{1.808}$	520	24	x	**1.10**
1_2-3	$Li_{5.844}PS_{4.868}Cl_{1.109}$	520	12	x	**2.14**
1_2-4	$Li_{6.998}PS_{5.203}Cl_{1.591}$	520	12	o	**1.18**
1_2-5	$Li_{9}PS_{5.734}Cl_{2.531}$	520	24	o	**0.77**
1_2-6	$Li_{7.992}PS_{5.811}Cl_{1.37}$	520	24	o	**1.13**
1_2-7	$Li_{6.288}PS_{4.991}Cl_{1.307}$	520	24	o	**1.55**
1_2-8	$Li_{4.204}PS_{4.183}Cl_{0.839}$	520	12	o	**0.11**
1_2-9	$Li_{6.288}PS_{4.991}Cl_{1.307}$	520	12	x	**1.50**
1_2-10	$Li_{6.262}PS_{5.023}Cl_{1.216}$	500	12	x	**1.53**
1_2-11	$Li_{6.929}PS_{5.231}Cl_{1.468}$	550	12	o	**1.26**
1_2-12	$Li_{5.435}PS_{4.58}Cl_{1.274}$	550	12	x	**2.54**
1_2-13	$Li_{5.640}PS_{4.759}Cl_{1.123}$	520	12	o	**1.96**
1_2-14	$Li_{5.837}PS_{4.725}Cl_{1.387}$	520	12	x	**2.86**
1_2-15	$Li_{5.674}PS_{4.725}Cl_{1.223}$	520	12	o	**2.56**
1_2-16	$Li_{5.793}PS_{4.72}Cl_{1.352}$	520	12	o	**1.73**
1_2-17	$Li_{5.924}PS_{4.817}Cl_{1.289}$	500	12	x	**1.34**
1_2-18	$Li_{6.22}PS_{4.994}Cl_{1.232}$	520	12	x	**1.87**

Sample No.	Composition	Temperature (℃)	Time (h)	Carbon paper	Ionic conductivity (mS・cm^{-1})
1_3-1	$Li_{5.284}PS_{4.473}Cl_{1.338}$	520	12	x	**2.51**
1_3-2	$Li_{5.326}PS_{4.495}Cl_{1.335}$	550	12	x	**2.95**
1_3-3	$Li_{5.082}PS_{4.4}Cl_{1.282}$	520	24	x	**1.00**
1_3-4	$Li_{5.383}PS_{4.522}Cl_{1.338}$	520	24	o	**1.56**
1_3-5	$Li_{4.972}PS_{4.463}Cl_{1.045}$	520	12	x	**1.51**
1_3-6	$Li_{4.63}PS_{4.143}Cl_{1.345}$	520	12	x	**0.21**
1_3-7	$Li_{5.239}PS_{4.445}Cl_{1.348}$	550	12	x	**1.79**
1_3-8	$Li_{6.906}PS_{5.088}Cl_{1.73}$	520	12	o	**1.59**
1_3-9	$Li_{6.354}PS_{4.923}Cl_{1.508}$	520	12	o	**1.83**
1_3-10	$Li_{5.204}PS_{4.384}Cl_{1.436}$	550	12	o	**1.64**
1_3-11	$Li_{6.412}PS_{4.987}Cl_{1.438}$	550	36	x	**0.72**
1_3-12	$Li_{5.948}PS_{4.748}Cl_{1.452}$	520	12	o	**2.02**
1_3-13	$Li_{5.606}PS_{4.733}Cl_{1.14}$	520	12	o	**1.85**
1_3-14	$Li_{5.837}PS_{4.725}Cl_{1.387}$	520	12	x	**2.86**
1_3-15	$Li_{5.674}PS_{4.712}Cl_{1.25}$	520	12	o	**2.19**
1_3-16	$Li_{5.977}PS_{4.778}Cl_{1.421}$	520	12	x	**2.24**
1_3-17	$Li_{6.307}PS_{4.922}Cl_{1.463}$	520	12	x	**0.84**
1_3-18	$Li_{7.125}PS_{5.242}Cl_{1.641}$	520	12	x	**1.07**

Sample No.	Composition	Temperature (℃)	Time (h)	Carbon paper	Ionic conductivity (mS ・ cm^{-1})
1_4-1	$Li_{5.362}PS_{4.511}Cl_{1.339}$	520	12	x	**2.01**
1_4-2	$Li_{5.326}PS_{4.495}Cl_{1.335}$	550	12	x	**3.00**
1_4-3	$Li_{5.766}PS_{4.604}Cl_{1.557}$	550	12	x	**1.93**
1_4-4	$Li_{5.379}PS_{4.52}Cl_{1.338}$	520	12	o	**1.60**
1_4-5	$Li_{6.098}PS_{4.82}Cl_{1.457}$	520	12	o	**2.30**
1_4-6	$Li_{4.181}PS_{4.071}Cl_{1.039}$	520	36	x	**0.01**
1_4-7	$Li_{5.198}PS_{4.438}Cl_{1.322}$	550	12	o	**2.32**
1_4-8	$Li_{5.04}PS_{4.456}Cl_{1.128}$	550	12	x	**2.46**
1_4-9	$Li_{5.558}PS_{4.629}Cl_{1.3}$	520	12	x	**2.85**
1_4-10	$Li_{5.574}PS_{4.618}Cl_{1.337}$	550	12	x	**2.98**
1_4-11	$Li_{5.385}PS_{4.446}Cl_{1.494}$	550	12	o	**2.28**
1_4-12	$Li_{4.535}PS_{4.214}Cl_{1.108}$	550	12	x	**0.36**
1_4-13	$Li_{5.169}PS_{4.359}Cl_{1.451}$	520	12	x	**2.31**
1_4-14	$Li_{5.7}PS_{4.348}Cl_{1.312}$	520	12	x	**1.84**
1_4-15	$Li_{4.96}PS_{4.306}Cl_{1.348}$	520	12	x	**1.14**
1_4-16	$Li_{4.905}PS_{4.305}Cl_{1.295}$	520	12	x	**0.63**
1_4-17	$Li_{4.815}PS_{4.28}Cl_{1.254}$	520	12	x	**0.44**
1_4-18	$Li_{5.445}PS_{4.477}Cl_{1.503}$	520	12	x	**2.66**

Sample No.	Composition	Temperature (℃)	Time (h)	Carbon paper	Ionic conductivity (mS・cm^{-1})
1_5-1	$Li_{5.207}PS_{4.44}Cl_{1.328}$	520	12	x	**1.47**
1_5-2	$Li_{5.326}PS_{4.495}Cl_{1.335}$	550	12	x	**2.91**
1_5-3	$Li_{4.264}PS_{4.296}Cl_{0.672}$	500	12	x	**0.65**
1_5-4	$Li_{5.34}PS_{4.502}Cl_{1.336}$	550	12	x	**1.86**
1_5-5	$Li_{5.221}PS_{4.478}Cl_{1.266}$	550	12	x	**1.22**
1_5-6	$Li_{5.387}PS_{4.487}Cl_{1.414}$	550	12	o	**2.41**
1_5-7	$Li_{5.334}PS_{4.496}Cl_{1.342}$	550	12	x	**2.50**
1_5-8	$Li_{5.017}PS_{4.401}Cl_{1.215}$	550	12	x	**1.95**
1_5-9	$Li_{5.201}PS_{4.457}Cl_{1.288}$	550	12	x	**2.31**
1_5-10	$Li_{5.572}PS_{4.608}Cl_{1.357}$	550	12	x	**2.70**
1_5-11	$Li_{5.51}PS_{4.501}Cl_{1.508}$	550	12	x	**3.89**
1_5-12	$Li_{7.354}PS_{5.236}Cl_{1.881}$	520	12	x	**1.18**
1_5-13	$Li_{5.257}PS_{4.419}Cl_{1.419}$	550	12	x	**1.32**
1_5-14	$Li_{5.556}PS_{4.595}Cl_{1.365}$	550	12	x	**1.33**
1_5-15	$Li_{4.729}PS_{4.295}Cl_{1.139}$	550	12	x	**0.79**
1_5-16	$Li_{7.452}PS_{5.449}Cl_{1.554}$	520	12	x	**1.52**
1_5-17	$Li_{6.905}PS_{5.26}Cl_{1.387}$	500	12	x	**1.99**
1_5-18	$Li_{5.463}PS_{4.588}Cl_{1.288}$	520	12	o	**3.42**

Sample No.	Composition	Oxygen (% in S)	Temperature (℃)	Ionic conductivity (mS・cm-1)
2_1-1	$Li_{5.589}PS_{4.162}O_{0.448}Cl_{0.469}Br_{0.06}I_{0.841}$	9.71	550	**0.01**
2_1-2	$Li_{5.128}PS_{4.161}O_{0.294}Cl_{0.047}Br_{1.099}I_{0.073}$	6.59	550	**0.5**
2_1-3	$Li_{4.595}PS_{3.866}O_{0.375}Cl_{0.569}Br_{0.35}I_{0.194}$	8.84	550	**0.31**
2_1-4	$Li_{5.922}PS_{4.078}O_{0.602}Cl_{0.662}Br_{0.375}I_{0.525}$	12.87	520	**0.22**
2_1-5	$Li_{5.355}PS_{4.027}O_{0.438}Cl_{0.057}Br_{0.159}I_{1.208}$	9.81	550	**0.01**
2_1-6	$Li_{5.123}PS_{4.138}O_{0.295}Cl_{0.552}Br_{0.588}I_{0.118}$	6.66	520	**0.79**
2_1-7	$Li_{5.09}PS_{4.049}O_{0.398}Cl_{0.354}Br_{0.692}I_{0.151}$	8.96	550	**1.15**
2_1-8	$Li_{6.099}PS_{4.324}O_{0.364}Cl_{1.066}Br_{0.267}I_{0.391}$	7.76	520	**1.18**
2_1-9	$Li_{5.567}PS_{4.065}O_{0.45}Cl_{1.402}Br_{0.126}I_{0.008}$	9.96	550	**4.20**
2_1-10	$Li_{5.104}PS_{3.814}O_{0.531}Cl_{0.259}Br_{0.483}I_{0.671}$	12.22	520	**0.02**
2_1-11	$Li_{4.936}PS_{4.176}O_{0.163}Cl_{0.083}Br_{0.787}I_{0.389}$	3.76	550	**0.05**
2_1-12	$Li_{4.434}PS_{3.892}O_{0.249}Cl_{0.62}Br_{0.044}I_{0.488}$	6.01	520	**0.02**
2_1-13	$Li_{5.572}PS_{4.09}O_{0.449}Cl_{0.259}Br_{1.004}I_{0.232}$	9.9	520	**0.8**
2_1-14	$Li_{5.901}PS_{4.411}O_{0.325}Cl_{0.945}Br_{0.302}I_{0.182}$	6.86	520	**1.42**
2_1-15	$Li_{5.181}PS_{4.043}O_{0.405}Cl_{0.547}Br_{0.67}I_{0.068}$	9.1	550	**2.84**
2_1-16	$Li_{4.91}PS_{4.112}O_{0.262}Cl_{0.392}Br_{0.368}I_{0.401}$	6	520	**0.14**
2_1-17	$Li_{6.033}PS_{4.622}O_{0.122}Cl_{0.555}Br_{0.954}I_{0.035}$	2.58	550	**1.56**
2_1-18	$Li_{5.339}PS_{3.601}O_{0.873}Cl_{1.158}Br_{0.043}I_{0.191}$	19.51	550	**0.49**

Sample No.	Composition	Oxygen (% in S)	Temperature (℃)	Ionic conductivity (mS · cm^{-1})
2_2-1	$Li_{5.194}PS_{3.918}O_{0.442}Cl_{1.2}Br_{0.027}I_{0.248}$	10.13	550	**0.27**
2_2-2	$Li_{6.058}PS_{4.208}O_{0.52}Cl_{0.408}Br_{1.193}$	11	520	**1.71**
2_2-3	$Li_{4.774}PS_{3.888}O_{0.384}Cl_{0.787}Br_{0.247}I_{0.195}$	9	550	**0.58**
2_2-4	$Li_{5.296}PS_{3.847}O_{0.543}Cl_{0.679}Br_{0.577}I_{0.259}$	12.36	520	**1.19**
2_2-5	$Li_{5.864}PS_{4.159}O_{0.463}Cl_{0.65}I_{0.971}$	10.02	550	**0.01**
2_2-6	$Li_{5.406}PS_{4.032}O_{0.456}Cl_{0.714}Br_{0.362}I_{0.355}$	10.17	520	**0.82**
2_2-7	$Li_{4.644}PS_{3.812}O_{0.408}Cl_{0.929}Br_{0.274}$	9.67	550	**0.78**
2_2-8	$Li_{5.392}PS_{3.95}O_{0.519}Cl_{1.42}Br_{0.022}I_{0.012}$	11.61	550	**2.04**
2_2-9	$Li_{5.567}PS_{4.065}O_{0.45}Cl_{1.402}Br_{0.126}I_{0.008}$	9.96	550	**4.28**
2_2-10	$Li_{5.344}PS_{3.959}O_{0.481}Cl_{0.576}Br_{0.624}I_{0.264}$	10.83	550	**0.65**
2_2-11	$Li_{5.041}PS_{3.764}O_{0.55}Cl_{1.073}Br_{0.251}I_{0.09}$	12.76	550	**0.88**
2_2-12	$Li_{4.852}PS_{3.927}O_{0.376}Cl_{1.189}Br_{0.057}$	8.74	550	**0.83**
2_2-13	$Li_{5.497}PS_{4.028}O_{0.449}Cl_{1.02}Br_{0.522}$	10.02	550	**1.84**
2_2-14	$Li_{5.219}PS_{4.018}O_{0.378}Cl_{1.319}Br_{0.109}$	8.61	550	**1.74**
2_2-15	$Li_{5.694}PS_{4.053}O_{0.457}Cl_{1.062}Br_{0.612}$	10.14	550	**1.91**
2_2-16	$Li_{5.543}PS_{4.219}O_{0.316}Cl_{1.349}Br_{0.125}$	6.97	550	**2.31**
2_2-17	$Li_{5.332}PS_{3.805}O_{0.596}Cl_{0.769}Br_{0.761}$	13.55	520	**1.28**
2_2-18	$Li_{5.596}PS_{3.988}O_{0.513}Cl_{1.394}Br_{0.021}I_{0.179}$	11.4	550	**0.38**

Sample No.	Composition	Oxygen (% in S)	Temperature (℃)	Ionic conductivity (mS・cm^{-1})
2_3-1	$Li_{5.535}PS_{4.062}O_{0.45}Cl_{1.236}Br_{0.274}$	9.98	550	**4.17**
2_3-2	$Li_{5.735}PS_{4.165}O_{0.432}Cl_{0.393}Br_{1.149}$	9.4	520	**2.38**
2_3-3	$Li_{5.186}PS_{3.986}O_{0.412}Cl_{1.098}Br_{0.216}I_{0.074}$	9.36	550	**2.72**
2_3-4	$Li_{5.332}PS_{4.012}O_{0.396}Cl_{1.348}Br_{0.167}$	8.98	550	**4.18**
2_3-5	$Li_{5.57}PS_{4.062}O_{0.45}Cl_{0.793}Br_{0.754}$	9.98	550	**3.79**
2_3-6	$Li_{5.717}PS_{4.092}O_{0.45}Cl_{1.579}Br_{0.054}$	9.9	520	**4.08**
2_3-7	$Li_{5.759}PS_{4.3}O_{0.43}Cl_{0.833}Br_{0.235}I_{0.23}$	9.09	520	**1.25**
2_3-8	$Li_{5.775}PS_{4.2}O_{0.384}Cl_{1.421}Br_{0.14}I_{0.045}$	8.37	520	**3.49**
2_3-9	$Li_{5.567}PS_{4.065}O_{0.45}Cl_{1.402}Br_{0.126}I_{0.008}$	9.96	550	**4.29**
2_3-10	$Li_{5.729}PS_{4.15}O_{0.422}Cl_{1.359}Br_{0.225}$	9.23	550	**4.02**
2_3-11	$Li_{5.683}PS_{4.078}O_{0.497}Cl_{1.174}Br_{0.265}I_{0.095}$	10.86	550	**3.36**
2_3-12	$Li_{6.043}PS_{4.16}O_{0.506}Cl_{1.578}Br_{0.119}I_{0.015}$	10.84	550	**2.58**
2_3-13	$Li_{5.572}PS_{4.064}O_{0.45}Cl_{1.227}Br_{0.027}I_{0.292}$	9.96	550	**0.91**
2_3-14	$Li_{6.156}PS_{4.311}O_{0.381}Cl_{1.112}Br_{0.399}I_{0.26}$	8.13	550	**2.18**
2_3-15	$Li_{4.985}PS_{3.892}O_{0.405}Cl_{0.732}Br_{0.359}I_{0.3}$	9.42	550	**1.07**
2_3-16	$Li_{5.247}PS_{3.897}O_{0.499}Cl_{1.328}Br_{0.118}I_{0.008}$	11.35	550	**3.1**
2_3-17	$Li_{5.683}PS_{4.466}O_{0.134}Cl_{1.477}I_{0.005}$	2.91	550	**2.64**
2_3-18	$Li_{4.674}PS_{3.413}O_{0.819}Cl_{1.103}Br_{0.042}I_{0.067}$	19.35	550	**0.98**

Sample No.	Composition	Oxygen (% in S)	Temperature (℃)	Ionic conductivity (mS・cm^{-1})
2_4-1	$Li_{5.513}PS_{4.048}O_{0.448}Cl_{1.246}Br_{0.276}$	9.97	550	**4.51**
2_4-2	$Li_{5.382}PS_{4.015}O_{0.416}Cl_{0.387}Br_{1.132}$	9.38	550	**1.88**
2_4-3	$Li_{5.197}PS_{3.969}O_{0.433}Cl_{1.107}Br_{0.192}I_{0.094}$	9.84	550	**2.43**
2_4-4	$Li_{5.728}PS_{4.148}O_{0.441}Cl_{1.379}Br_{0.171}$	9.6	550	**2.79**
2_4-5	$Li_{5.583}PS_{4.068}O_{0.451}Cl_{0.792}Br_{0.753}$	9.97	550	**4.24**
2_4-6	$Li_{5.513}PS_{4.057}O_{0.448}Cl_{1.453}Br_{0.05}$	9.95	550	**3.96**
2_4-7	$Li_{6.154}PS_{4.165}O_{0.483}Cl_{1.745}Br_{0.114}$	10.4	550	**2.79**
2_4-8	$Li_{5.504}PS_{4.039}O_{0.453}Cl_{1.384}Br_{0.116}I_{0.02}$	10.08	550	**3.75**
2_4-9	$Li_{5.567}PS_{4.064}O_{0.451}Cl_{1.402}Br_{0.126}I_{0.008}$	9.98	550	**4.26**
2_4-10	$Li_{5.535}PS_{4.06}O_{0.442}Cl_{1.313}Br_{0.218}$	9.81	550	**4.12**
2_4-11	$Li_{5.537}PS_{4.05}O_{0.456}Cl_{1.416}Br_{0.11}$	10.12	550	**3.26**
2_4-12	$Li_{5.554}PS_{4.065}O_{0.442}Cl_{1.417}Br_{0.122}$	9.8	550	**3.6**
2_4-13	$Li_{5.442}PS_{4.013}O_{0.446}Cl_{1.181}Br_{0.342}$	10.01	550	**4.36**
2_4-14	$Li_{5.673}PS_{4.099}O_{0.451}Cl_{1.137}Br_{0.279}I_{0.157}$	9.91	550	**2.14**
2_4-15	$Li_{5.041}PS_{3.964}O_{0.421}Cl_{0.91}Br_{0.361}$	9.61	550	**3.89**
2_4-16	$Li_{5.713}PS_{4.113}O_{0.454}Cl_{1.442}Br_{0.129}I_{0.009}$	9.93	550	**3.7**
2_4-17	$Li_{5.616}PS_{4.168}O_{0.39}Cl_{1.326}Br_{0.154}I_{0.02}$	8.56	550	**3.44**
2_4-18	$Li_{6.038}PS_{4.275}O_{0.394}Cl_{1.549}Br_{0.058}I_{0.093}$	8.43	520	**2.37**

Sample No.	Composition	Oxygen (% in S)	Temperature (℃)	Ionic conductivity (mS・cm^-1^)
2_5-1	$Li_{5.513}PS_{4.048}O_{0.448}Cl_{1.246}Br_{0.276}$	9.97	550	**4.34**
2_5-2	$Li_{5.512}PS_{4.073}O_{0.422}Cl_{0.899}Br_{0.622}$	9.38	550	**5.24**
2_5-3	$Li_{5.401}PS_{4.021}O_{0.439}Cl_{1.185}Br_{0.296}$	9.84	550	**4.94**
2_5-4	$Li_{5.334}PS_{3.988}O_{0.424}Cl_{1.132}Br_{0.363}I_{0.015}$	9.6	550	**3.49**
2_5-5	$Li_{5.498}PS_{4.493}Cl_{0.835}Br_{0.676}$	0	550	**6.9**
2_5-6	$Li_{5.787}PS_{4.119}O_{0.455}Cl_{1.161}Br_{0.369}I_{0.108}$	9.95	520	**3.51**
2_5-7	$Li_{6.025}PS_{4.199}O_{0.487}Cl_{1.398}Br_{0.217}I_{0.038}$	10.4	550	**2.56**
2_5-8	$Li_{5.512}PS_{4.043}O_{0.453}Cl_{1.378}Br_{0.143}$	10.08	550	**3.57**
2_5-9	$Li_{5.484}PS_{4.039}O_{0.448}Cl_{1.128}Br_{0.309}I_{0.072}$	9.98	550	**3.51**
2_5-10	$Li_{5.503}PS_{4.052}O_{0.441}Cl_{1.221}Br_{0.282}I_{0.014}$	9.81	550	**4.48**
2_5-11	$Li_{5.571}PS_{4.068}O_{0.458}Cl_{0.629}Br_{0.669}I_{0.221}$	10.12	550	**2.62**
2_5-12	$Li_{5.472}PS_{4.044}O_{0.439}Cl_{1.213}Br_{0.272}I_{0.021}$	9.8	550	**3.97**
2_5-13	$Li_{5.536}PS_{4.054}O_{0.451}Cl_{1.183}Br_{0.342}$	10.01	550	**4.57**
2_5-14	$Li_{5.553}PS_{4.061}O_{0.447}Cl_{1.003}Br_{0.533}$	9.91	550	**3.78**
2_5-15	$Li_{5.488}PS_{4.055}O_{0.431}Cl_{1.085}Br_{0.431}$	9.61	550	**4.89**
2_5-16	$Li_{5.412}PS_{4.018}O_{0.443}Cl_{1.297}Br_{0.192}$	9.93	550	**3.91**
2_5-17	$Li_{5.505}PS_{4.108}O_{0.385}Cl_{1.198}Br_{0.321}$	8.56	550	**4.82**
2_5-18	$Li_{5.747}PS_{4.184}O_{0.385}Cl_{1.234}Br_{0.346}I_{0.029}$	8.43	550	**3.8**

Sample No.	Composition	Oxygen (% in S)	Temperature (℃)	Ionic conductivity (mS・cm⁻¹)
2_6-1	$Li_{5.479}PS_{4.486}Cl_{0.838}Br_{0.635}I_{0.035}$	0	550	**6.26**
2_6-2	$Li_{5.525}PS_{4.503}Cl_{0.888}Br_{0.612}$	0	550	**7.78**
2_6-3	$Li_{5.514}PS_{4.495}Cl_{0.96}Br_{0.563}$	0	550	**6.73**
2_6-4	$Li_{5.514}PS_{4.221}O_{0.278}Cl_{1.148}Br_{0.369}$	6.19	550	**6.19**
2_6-5	$Li_{5.498}PS_{4.493}Cl_{0.835}Br_{0.676}$	0	550	**6.93**
2_6-6	$Li_{5.501}PS_{4.052}O_{0.449}Cl_{0.832}Br_{0.657}$	9.97	550	**5.53**
2_6-7	$Li_{6.02}PS_{4.607}Cl_{1.182}Br_{0.469}I_{0.154}$	0	550	**4.1**
2_6-8	$Li_{5.466}PS_{4.481}Cl_{0.758}Br_{0.545}I_{0.201}$	0	550	**3.47**
2_6-9	$Li_{5.588}PS_{4.522}Cl_{1.086}Br_{0.458}$	0	550	**5.97**
2_6-10	$Li_{5.505}PS_{4.390}O_{0.105}Cl_{1.211}Br_{0.303}$	2.33	550	**6.34**
2_6-11	$Li_{5.564}PS_{4.521}Cl_{1.276}Br_{0.246}$	0	550	**5.82**
2_6-12	$Li_{5.493}PS_{4.255}O_{0.237}Cl_{0.634}Br_{0.827}I_{0.048}$	5.28	550	**5.39**
2_6-13	$Li_{5.495}PS_{4.492}Cl_{0.743}Br_{0.669}I_{0.098}$	0	550	**4.92**
2_6-14	$Li_{5.558}PS_{4.214}O_{0.297}Cl_{0.756}Br_{0.773}I_{0.006}$	6.59	550	**6.53**
2_6-15	$Li_{5.508}PS_{4.499}Cl_{0.849}Br_{0.615}I_{0.047}$	0	550	**6.91**
2_6-16	$Li_{5.471}PS_{4.130}O_{0.353}Cl_{1.312}Br_{0.194}$	7.88	550	**5.03**
2_6-17	$Li_{5.495}PS_{4.494}Cl_{0.971}Br_{0.505}I_{0.031}$	0	550	**6.97**
2_6-18	$Li_{5.58}PS_{4.519}Cl_{0.748}Br_{0.795}$	0	550	**7.11**
